# Supplementary material for: Safety and immunogenicity of a SARS-CoV-2 Gamma variant RBD-based protein adjuvanted vaccine used as booster in healthy adults
Source: Nat Commun. 2023 Jul 28;14:4551. doi: 10.1038/s41467-023-40272-3 (PMC10382514; doi:10.1038/s41467-023-40272-3)
Supplement: Supplementary file 1 — Supplementary Information [file 41467_2023_40272_MOESM1_ESM.pdf]

Supplementary Figures, Tables and Methods

Supplementary Figures

Supplementary Figure 1

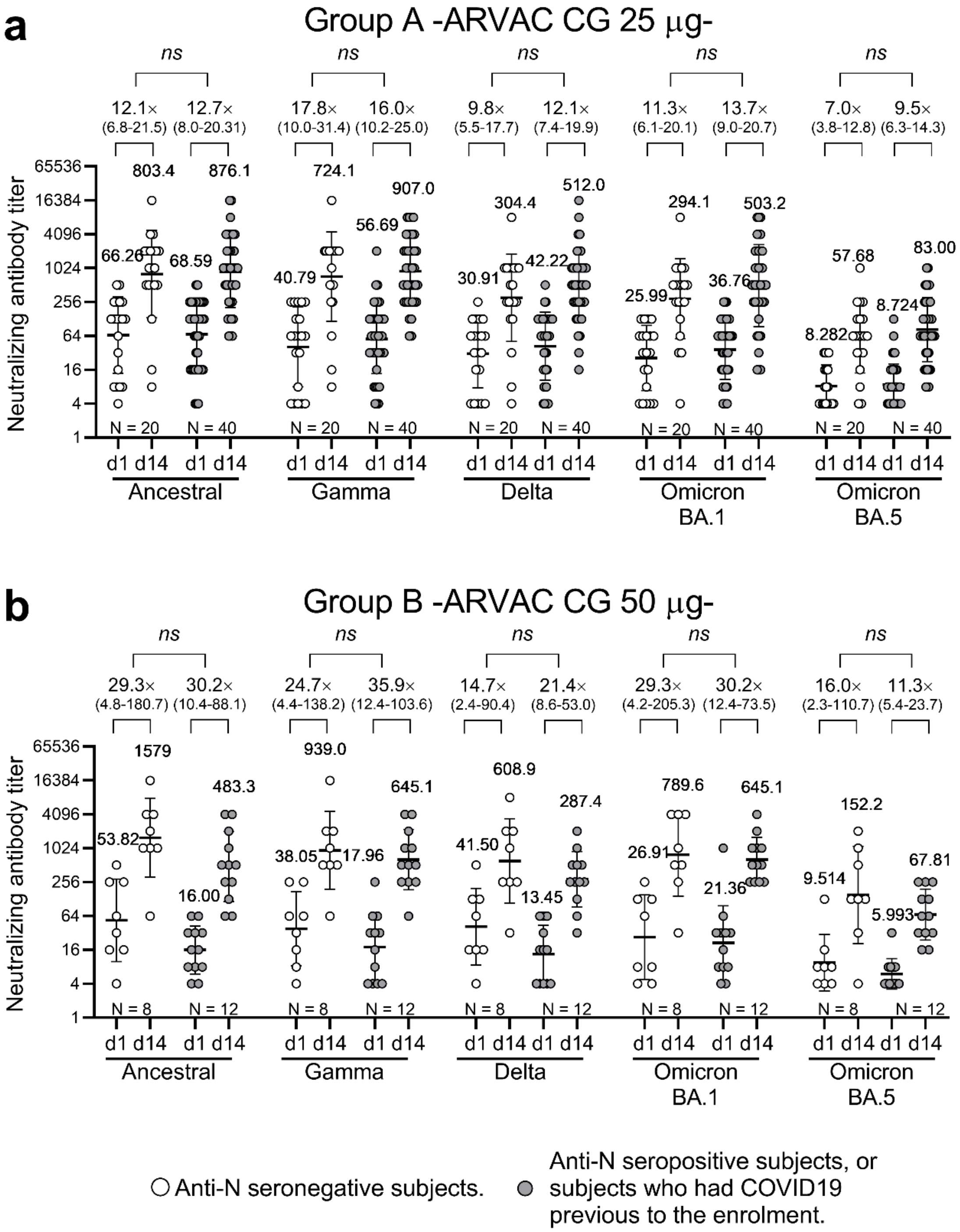

Supplementary Figure 1: Administration of ARVAC CG as booster increases the Neutralizing antibody titers against the Ancestral, Gamma, Delta, Omicron BA.1 and Omicron BA.5 variants of SARS-CoV-2 irrespective of the previous history of COVID19 or anti-N serology of the study participants. Study participants were classified according to their previous story of COVID19 and/or their anti-N serology into two

groups: in the graphs are shown with open circles those who were seronegative for anti-N IgG (at day 1 and at day 28) and have no previous diagnostic of COVID19 (N = 20 in Group A and N = 8 in group B) and shown with filled circles are those who were seropositive for anti-N IgG (at day 1 or at day 28) or have had COVID19 previous to the study (N = 40 in Group A and N = 12 in group B). The neutralizing antibody titers against the Ancestral, Gamma, Delta and Omicron BA.1 and Omicron BA.5 variants of SARS-CoV-2 in plasma samples of individuals boosted with ARVAC CG 25µg (a) or 50 µg (b) prior to the vaccine administration (d1) or after 14 days of booster administration (d14) are shown. Each point represents the nAb titer of a volunteer at the indicated time point and against the depicted viral variant. The nAb geometric mean titers (GMTs) with 95% CIs are shown as horizontal and error bars, respectively. The numbers depicted above the individual points for each specified subgroup, time point and viral variant represent the GMT. The fold increases in the GMT from day 1 to day 14 (GMFR) for each specified subgroup of participants and variant are shown with a number followed by a × with the 95% CI written below between brackets. The number of participants included in each data set analyzed is depicted in the bottom of the bar (N = number of individuals in each data set). Statistical differences were analyzed using the two-tailed Mann Whitney test. ns: P>0.05.

Supplementary Figure 2

Neutralizing antibody responses in individuals with BBIBP-CorV primary vaccination stratified by anti-N serology and/or previous COVID19 history.

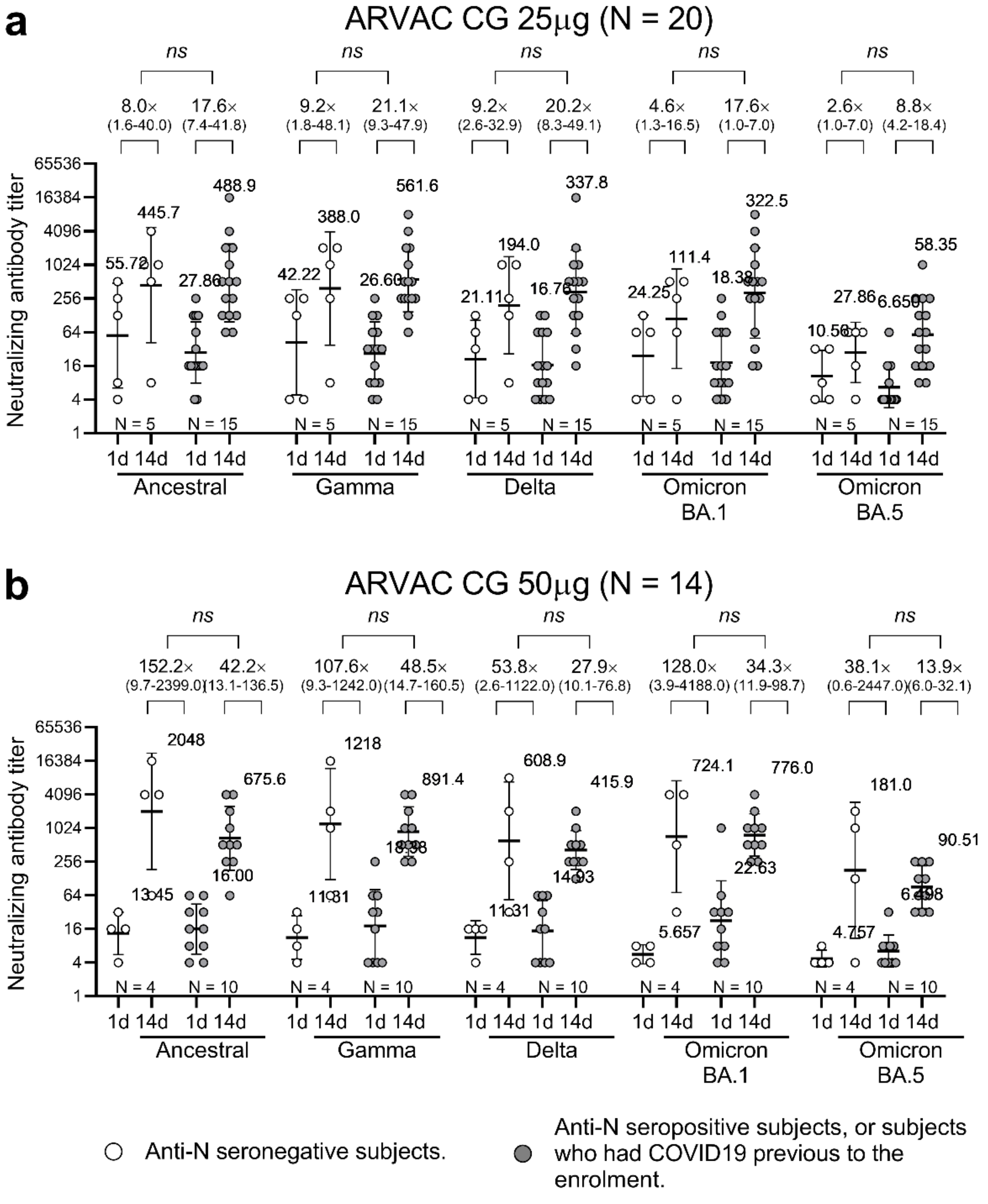

Supplementary Figure 2: Administration of ARVAC CG as booster to individuals previously vaccinated with BBIBP-CorV vaccine increases the neutralizing antibody titers against the Ancestral, Gamma, Delta, Omicron BA.1 and Omicron BA.5 variants of SARS-CoV-2 irrespective of the previous history of COVID19 or anti-N serology of the study participants. Study participants with a primary vaccination scheme with the BBIBP-CorV vaccine were classified according to their previous story of COVID-19 and/or their anti-N serology into two groups: in the graphs are shown with open circles those who were seronegative for anti-N IgG (at day 1 and at day 28) and have no previous diagnostic of COVID-19 (*N* = 5 in Group A and *N* = 4 in

group B) and shown with filled circles are those who were seropositive for anti-N IgG (at day 1 or at day 28) or have had COVID19 previous to the study (*N* = 15 in Group A and *N* = 10 in group B). The neutralizing antibody titers against the Ancestral, Gamma, Delta and Omicron BA.1 and Omicron BA.5 variants of SARS-CoV-2 in plasma samples of individuals boosted with ARVAC CG 25µg (a) or 50 µg (b) prior to the vaccine administration (d1) or after 14 days of booster administration (d14) are shown. Each point represents the nAb titer of a volunteer at the indicated time point and against the depicted viral variant. The nAb geometric mean titers (GMTs) with 95% CIs are shown as horizontal and error bars, respectively. The numbers depicted above the individual points for each specified subgroup, time point and viral variant represent the GMT. The fold increases in the GMT from day 1 to day 14 (GMFR) for each specified subgroup of participants and variant are shown with a number followed by a × with the 95% CI written below between brackets. The number of participants included in each data set analyzed is depicted in the bottom of the bar (*N* = number of individuals in each data set). Statistical differences were analyzed using the two-tailed Mann Whitney test. ns: P>0.05.

Supplementary Figure 3

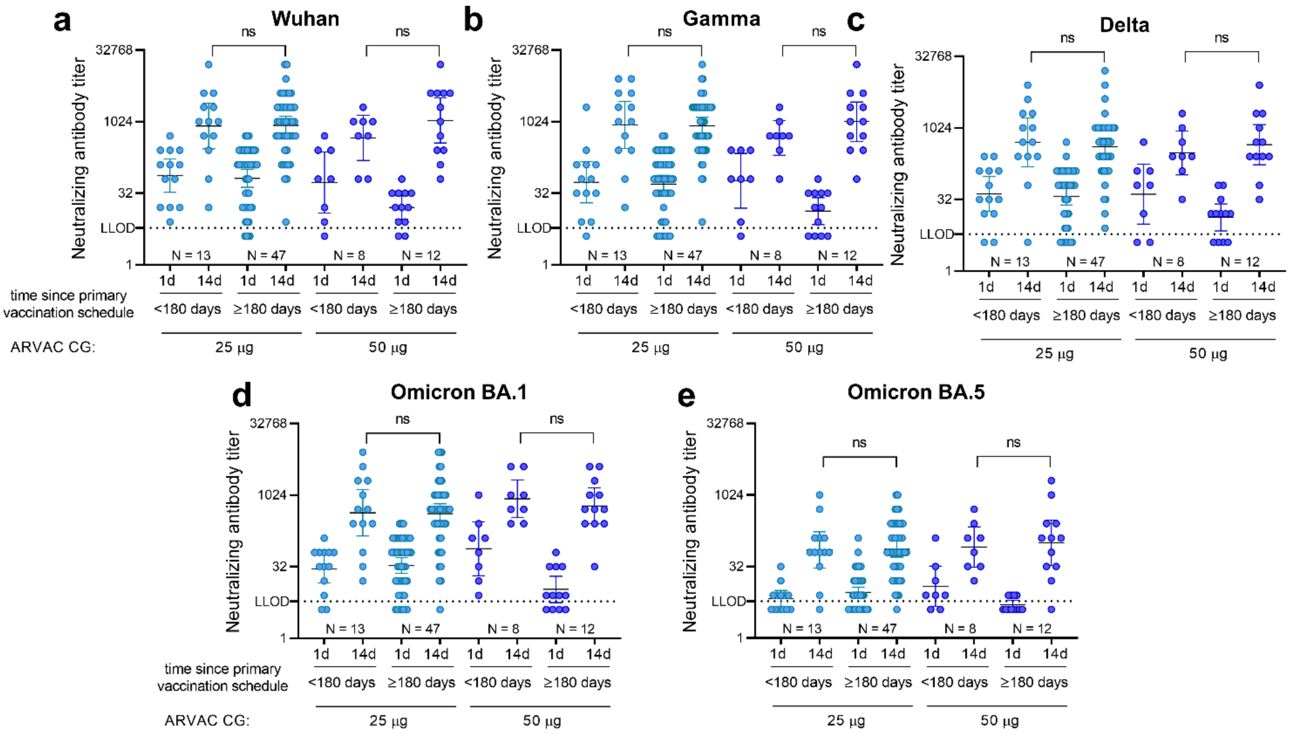

Supplementary Figure 3: Administration of ARVAC CG as booster increases the Neutralizing antibody titers against the Ancestral, Gamma, Delta, Omicron BA.1 and Omicron BA.5 variants of SARS-CoV-2 irrespective of the time since primary vaccination series completion. Study participants were classified according to the time since primary vaccination series completion in two groups: i) those with time since primary vaccination completion less than 180 days (N = 13 in Group A and N = 8 in group B) and ii) those with a time equal or greater than 180 days (N = 47 in Group A and N = 12 in group B). The neutralizing antibody titers against the Ancestral (a), Gamma (b), Delta (c) and Omicron BA.1 (d) and Omicron BA.5 (e) variants of SARS-CoV-2 in plasma samples of individuals boosted with ARVAC CG 25µg or 50 µg prior to the vaccine administration (1d) or after 14 days of booster administration (14d) are shown. Each point represents the nAb titer of a volunteer at the indicated time point and against the depicted viral variant. The nAb geometric mean titers (GMTs) with 95% CIs are shown as horizontal and error bars, respectively. The number of participants included in each data set analyzed is depicted in the bottom of the bar (N = number of individuals in each data set). Statistical differences were analyzed using the two-tailed Mann Whitney test. ns: P>0.05.

Supplementary Figure 4

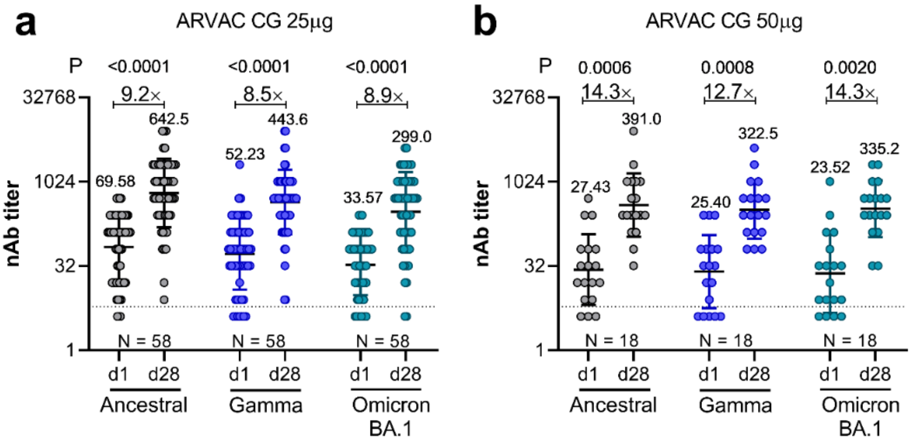

Supplementary Figure 4: Administration of ARVAC CG as booster increases after 28 days the nAb titers against the Ancestral, Gamma and Omicron BA.1 variants of SARS-CoV-2. Neutralizing antibody titers against the Ancestral, Gamma, and Omicron BA.1 variants of SARS-CoV-2 in plasma samples of individuals boosted with ARVAC CG 25µg (*N* = 58) (A) or 50 µg (*N* = 18) (B) prior to the vaccine administration (d1) or after 28 days of booster administration (d28). Each point represents the nAb titer of a volunteer at the indicated time point and against the depicted viral variant. The nAb geometric mean titers (GMT) with 95% CIs are shown as horizontal and error bars, respectively. The numbers depicted above the individual points for each specified time point and viral variant represent the GMT. The fold increases in the GMT from day 1 to day 28 (GMFR) for each specified variant are shown with a number followed by a ×. The dashed line represents the positivity threshold on the neutralization assay. The number of participants included in each data set analyzed is depicted in the bottom of the bar (*N* = number of individuals in each data set). Statistical differences were analyzed using the two-tailed Wilcoxon pair-matched test. *P* values are depicted above the data sets that were compared. *P* values are **(a)** *P*<10e-15 (Ancestral), *P*=0.000000000005 (Gamma), *P*=0.000000000000005 (Omicron BA.1), (b) *P*=0.0006 (Ancestral), *P*=0.0008 (Gamma), *P*=0.0020 (Omicron BA.1).

Supplementary Figure 5

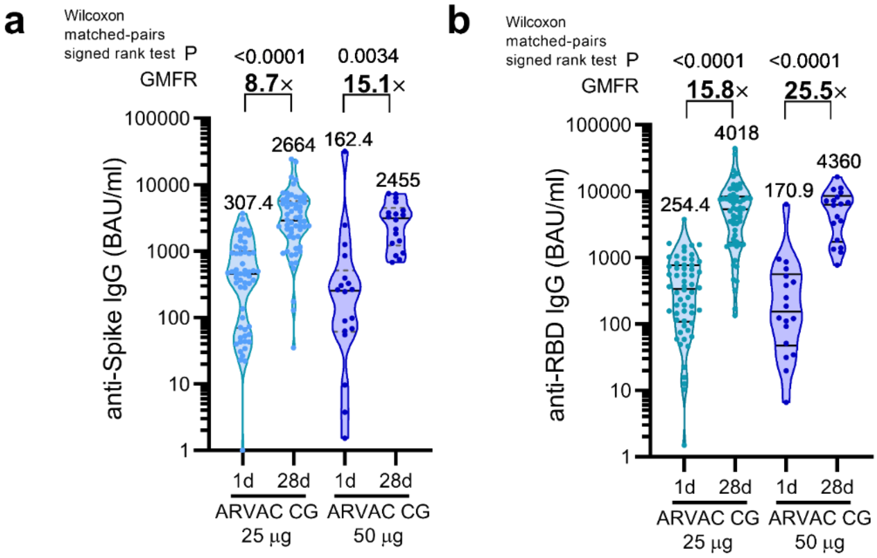

Supplementary Figure 5. Analysis of binding antibody response induced by ARVAC CG booster dose. (A) Serum anti-spike IgG (A) or anti-RBD IgG (B) were analyzed by ELISA. Antibody levels are expressed in BAU/ml according to the WHO International Antibody Standard. Graphs display violin plots showing the frequency distribution of the data and dots show individual values for each volunteer at a specified time point (*N* = 58 for ARVAC CG 25 µg cohort and *N* = 18 for ARVAC CG 50 µg cohort). The geometric means are shown above the violin plots. The fold raise in GM (GMFR) after 28 days of booster (d28) respect to baseline (d1) is shown above a line connecting both time points. The two-tailed Wilcoxon matched pairs test was used for statistical analysis. *P* values are depicted on the graph. *P* values: **(a)** *P*<10e-15 (ARVAC CG 25µg), *P*=0.0034 (ARVAC CG 50µg); **(b)** *P*<10e-15 (ARVAC CG 25µg), *P*=0.000015 (ARVAC CG 50µg).

Supplementary Figure 6

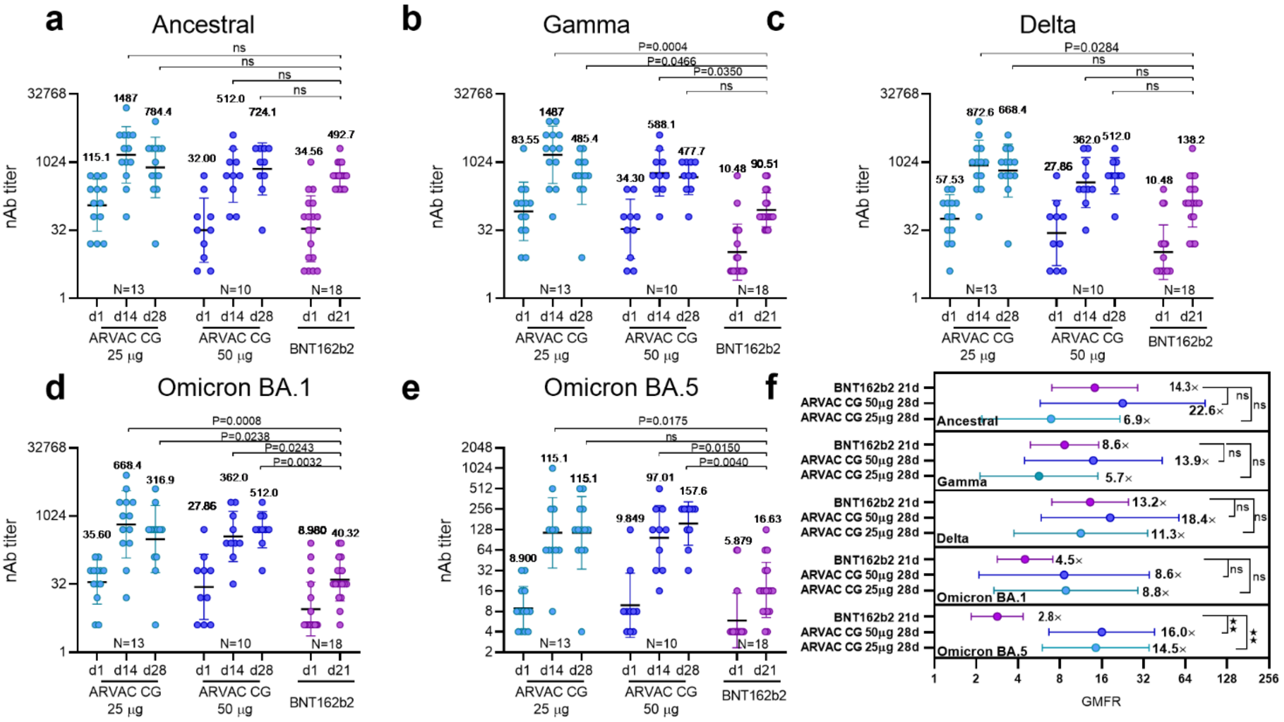

Supplementary Figure 6: Comparison of nAb GMT and GMFR after booster with ARVAC CG or booster with BNT162b2 in individuals whose time since completion of primary vaccination series was less than 180 days. Neutralizing antibody titers against the Ancestral **(a)**, Gamma **(b)**, Delta **(c)**, Omicron BA.1 **(d)** and Omicron BA.5 **(e)** variants of SARS-CoV-2 in plasma samples of individuals whose time since completion of primary vaccination series to booster was less than 180 days boosted with the indicated vaccine (ARVAC CG 25µg, ARVAC CG 50 µg or BNT162b2) prior to the booster administration (d1) or at the indicated days after booster (d14, d21 or d28). Each point represents the nAb titer of a volunteer boosted with the indicated vaccine, at the indicated time point and against the depicted viral variant. The nAb GMTs and 95% CIs are shown as horizontal and error bars, respectively. The numbers depicted above the individual points for each specified time point and viral variant represent the GMTs. The number of participants included in each data set analyzed is depicted in the bottom of the bar (*N* = number of individuals in each data set). Data are from participants whose time since completion of primary vaccination series to booster was less than 180 days and with no missing data at baseline and at all time points analyzed (ARVAC CG 25µg (*N* = 13), ARVAC CG 50 µg (*N* = 10) or BNT162b2 (*N* = 18). Statistical differences were analyzed using the Kruskal-Wallis test followed by the Dunn´s multiple comparison test. Exact *P* values are depicted above the data sets that were compared. ns: *P*>0.05 **(F)** Fold increases in the GMT from day 1 to day 21 or 28 (GMFR) for each specified variant represented by a point and written with a number followed by a ×. The horizontal lines represent the 95% CIs. Data are from participants whose time since completion of primary vaccination series to booster was less than 180 days and with no missing data at baseline and at all time points analyzed (ARVAC CG 25µg (*N* = 13), ARVAC CG 50 µg (*N* = 10) or BNT162b2 (*N* = 18). Statistical differences were analyzed using Kruskal-Wallis test followed by the Dunn´s multiple comparison test. ns: *P*>0.05; ★★: *P*<0.01. In Omicron BA.5 VOCs panel **(f)** *P* values are VOC: ARVAC CG 25µg vs. BNT162b2, *P*=0.003; ARVAC CG 50µg vs. BNT162b2, *P*=0.004.

Supplementary Figure 7

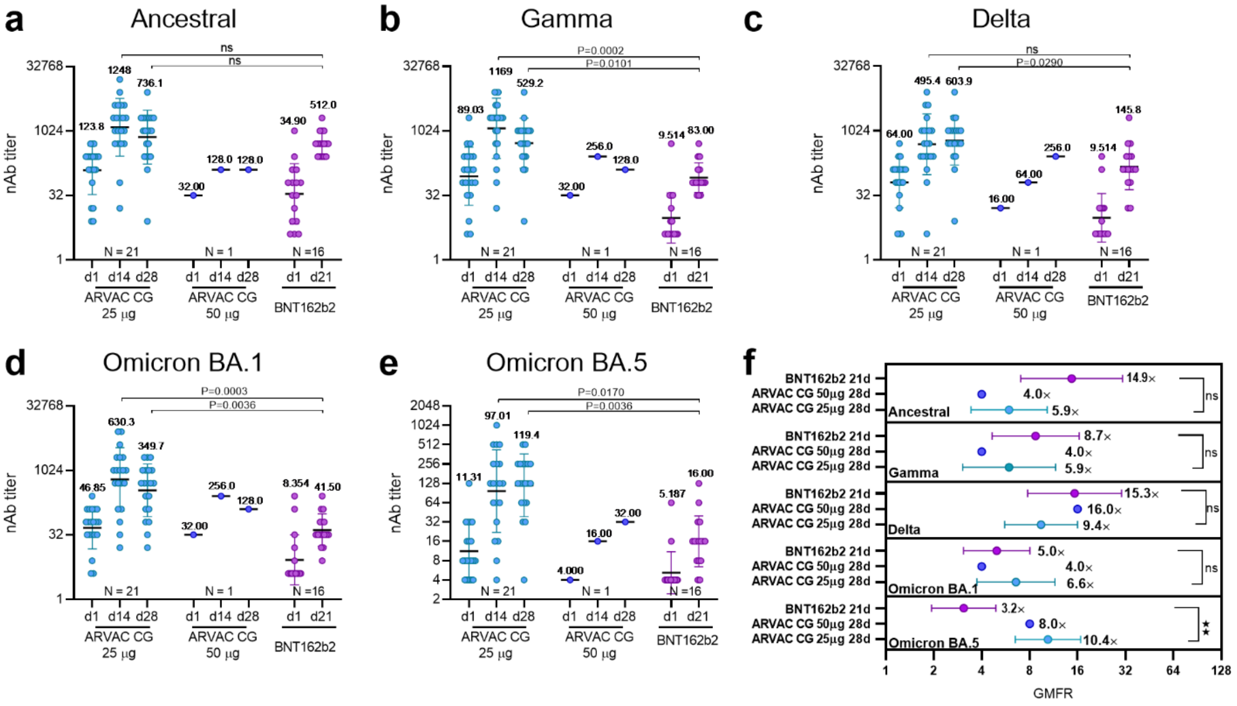

Supplementary Figure 7: Comparison of nAb GMT and GMFR after booster with ARVAC CG or booster with BNT162b2 in individuals whose primary vaccination was rAd26/rAd5. Neutralizing antibody titers against the Ancestral **(a)**, Gamma **(b)**, Delta **(c)**, Omicron BA.1 **(d)** and Omicron BA.5 **(e)** variants of SARS-CoV-2 in plasma samples of individuals whose primary vaccination was rAd26/rAd5 (Sputnik V vaccine) boosted with the indicated vaccine (ARVAC CG 25µg, ARVAC CG 50 µg or BNT162b2) prior to the booster administration (d1) or at the indicated days after booster (d14, d21 or d28). Each point represents the nAb titer of a volunteer boosted with the indicated vaccine, at the indicated time point and against the depicted viral variant. Data are from participants whose primary vaccination series was rAd26/rAd5 (ARVAC CG 25µg (N=21), ARVAC CG 50 µg (N=1) or BNT162b2 (N=16). The nAb GMTs and 95% CIs are shown as horizontal and error bars, respectively. The numbers depicted above the individual points for each specified time point and viral variant represent the GMTs. The number of participants included in each data set analyzed is depicted in the bottom of the bar (*N* = number of individuals in each data set). Statistical differences were analyzed using the Kruskal-Wallis test followed by the Dunn´s multiple comparison test. Exact *P* values are depicted above the data sets that were compared. ns: $P>0.05$ **(F)** Fold increases in the GMT from day 1 to day 21 or 28 (GMFR) for each specified variant is represented by a point and written with a number followed by a ×. The horizontal lines represent the 95% CIs. Data are from participants whose primary vaccination series was rAd26/rAd5 (ARVAC CG 25µg (N=21), ARVAC CG 50 µg (N=1) or BNT162b2 (*N* = 16). Statistical differences were analyzed using Kruskal-Wallis test followed by the Dunn´s multiple comparison test. ns: $P>0.05$, ★★: $P=0.0011$.

Supplementary Figure 8

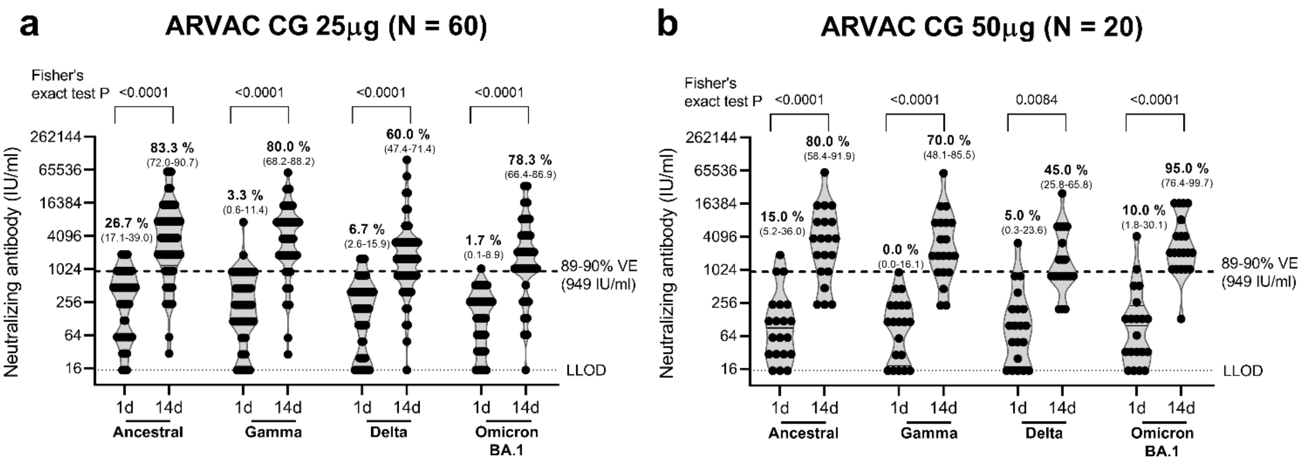

Supplementary Figure 8: Administration of a booster dose of ARVAC CG increases the frequency of individuals with nAb levels that correlate with high VE. The nAb titers prior and after the booster were transformed to international units per ml (IU/ml) by the inclusion in each plate of a secondary standard that was calibrated with the WHO international standard (NIBSC code: 20/268). A cut off value 949 IU/ml was used to determine the proportion of individuals with nAb levels ≥ 949 IU/ml (levels associated with 80-90% VE) prior to the booster administration (d1) or 14 days after administration (d14). Each point represents the nAb level (IU/ml) of a volunteer at the indicated time point and against the depicted viral variant (*N* = 60 for ARVAC CG 25μg and *N* = 20 for ARVAC CG 50 μg cohorts. The geometric mean of nAb levels with 95% CIs are shown as horizontal and error bars, respectively. The numbers depicted above the individual points for each specified time point and viral variant represent the percentage of individuals with nAb levels ≥ 949 IU/ml and the respective 95% CI. The dashed line represents the positivity threshold on the Neutralization assay (LLOD). Statistical differences were analyzed using the two-sided Fisher´s exact test. *P* Values are depicted above the data sets that were compared. Exact *P* values are: **(a)** *P*=0.0000000005 (Ancestral), *P*<10e-15 (Gamma and Omicron BA.1), *P*=0.0000000003 (Delta); **(b)** *P*=0.00009 (Ancestral), *P*=0.000003 (Gamma), *P*=0.0084 (Delta), *P*=0.00000006 (Omicron BA.1).

Supplementary Figure 9

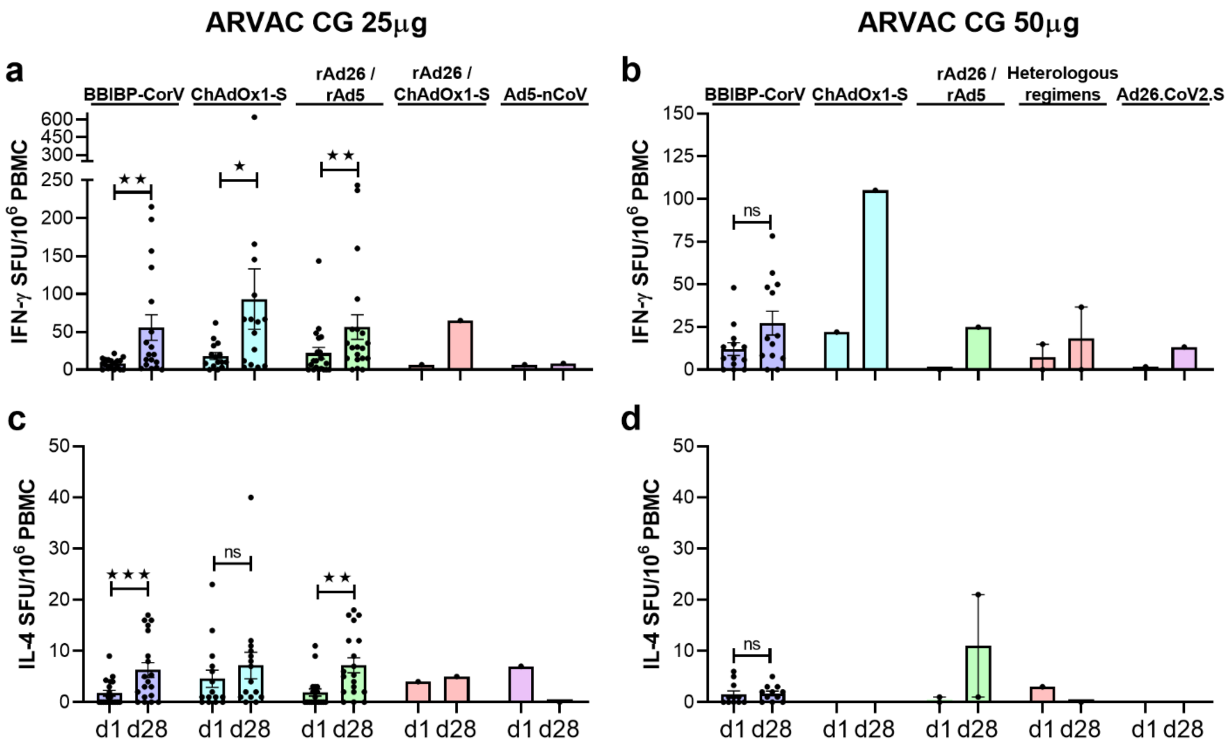

Supplementary Figure 9. ARVAC CG booster induces significant increase of Th1-predominant cell response measured by IFN-γ and IL-4 ELISpot after restimulation of PBMCs with RBD spanning peptide pool in individuals previously vaccinated with different primary vaccination schemes. Before booster administration (1d) and after 28 days (d28) of administration of ARVAC CG 25 µg (A, C) or 50 µg (B, D) dose, RBD-specific cellular responses were measured by IFN-γ (A, B) and IL-4 (C, D) ELISpot in PBMCs. Shown are spot-forming units (SFU) per 1 × 10^6 PBMCs producing IFN-γ and IL-4 after stimulation with-RBD peptide pool from samples with viable cells. Participants in each cohort were grouped according the received primary vaccination scheme. Group A: BBIBP-CorV (N = 18), ChAdOx1-S (N = 15), rAd26/rAd5 (N = 20), rAd26/ChAdOx1-S (heterologous vaccination, N = 1) and Ad5-nCoV (N = 1). Group B: BBIBP-CorV (N = 14), ChAdOx1-S (N = 1), rAd26/rAd5 (N = 1), heterologous vaccination regimens (ChAdOx1-S / mRNA1273 or BBIBP-CorV / BNT162b2; N = 3). And Ad26.CoV2.S (N = 1), . Each point represents the cytokine spot forming units (SFU) at the indicated time point. The SFU mean is represented by bars and SEM by error bars, respectively. Statistical differences were analyzed using the two-tailed Wilcoxon pair-matched test. ns: P>0.05; ⋆: P<0.05; ⋆⋆: P<0.01, ⋆⋆⋆: P<0.001. Exact P values are: **(a)** P=0.0093 (BBIBP-CorV), P=0.0130 (ChAdOx1-S), P=0.0041 (rAd26/rAd5); **(b)** P>0.05 (BBIBP-CorV); **(c)** P=0.0004 (BBIBP-CorV), P>0.05 (ChAdOx1-S), P=0.0014 (rAd26/rAd5); **(d)** P>0.05 (BBIBP-CorV).

Supplementary Figure 10

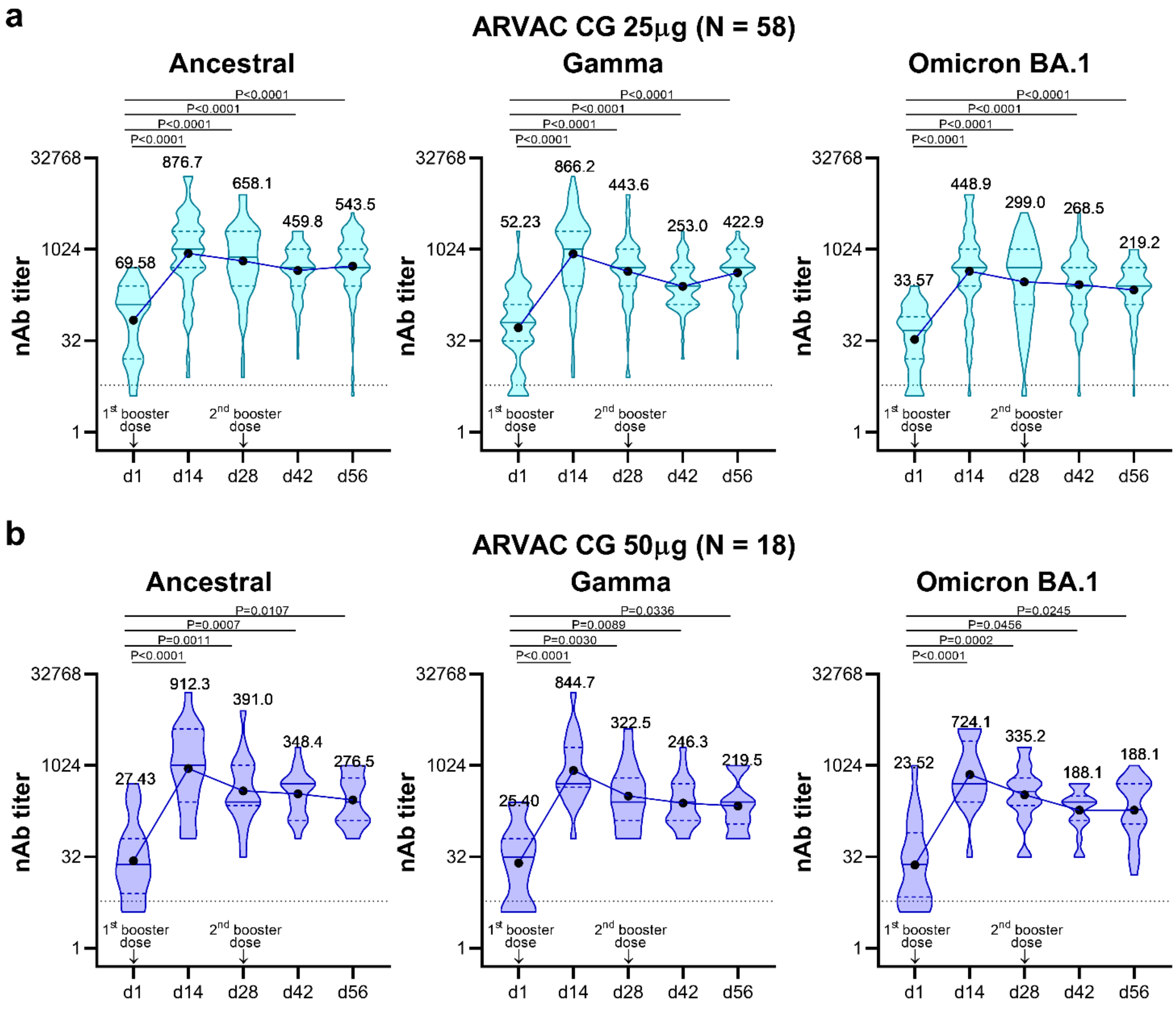

Supplementary Figure 10: The nAb titers against the Ancestral, Gamma and Omicron BA.1 variants of SARS-CoV-2 after the first and the second dose of the study. Neutralizing antibody titers against the Ancestral, Gamma, and Omicron BA.1 variants of SARS-CoV-2 in plasma samples of individuals boosted with ARVAC CG 25µg (A) or 50 µg (B) prior to the vaccine administration (d1) or after 14 or 28 days of first booster administration (d14 and d28) or second booster administration (d42 and d56). Violin plots represent the frequency distribution of data of the volunteers at the indicated time point and against the depicted viral variant. Lines inside each violin plot indicate the median (solid line) and quartiles (dashed lines). The geometric mean of nAb titers (GMT) are written above the violin points and represented as points connected by a line along different time points. The dashed line represents the positivity threshold on the neutralization assay. Statistical differences were analyzed using the Friedman test (non-parametric paired ANOVA) followed by two-sided Dunn´s multiple comparison test. *P* Values are depicted above the data sets that were compared. Exact *P* values vs. d1 are: **(a)** Ancestral strain: *P*<10e-15 (d14), *P*=0.000000000000002 (d28), *P*=0.0000000070 (d42), *P*= 0.00000000000055 (d56); Gamma VOC: *P*<10e-15 (d14), *P*=0.0000000000004 (d28), *P*=0.00007 (d42), *P*=0.0000000000011 (d56); Omicron BA.1: *P*<10e-15 (d14), *P*=0.000000000000011 (d28), *P*=0.0000000000004 (d42), *P*=0.000000006 (d56). **(b)** Ancestral strain: *P*=0.00000001 (d14), *P*=0.0011 (d28), *P*=0.0007 (d42), *P*= 0.0107 (d56); Gamma VOC: *P*=0.0000000020 (d14), *P*=0.0030 (d28), *P*=0.0089 (d42), *P*=0.0336 (d56); Omicron BA.1: *P*=0.000000003 (d14), *P*=0.0002 (d28), *P*=0.0456 (d42), *P*=0.0245 (d56). Arrows indicate the days of first booster (1d) and second booster administration (28d).

Supplementary Figure 11.

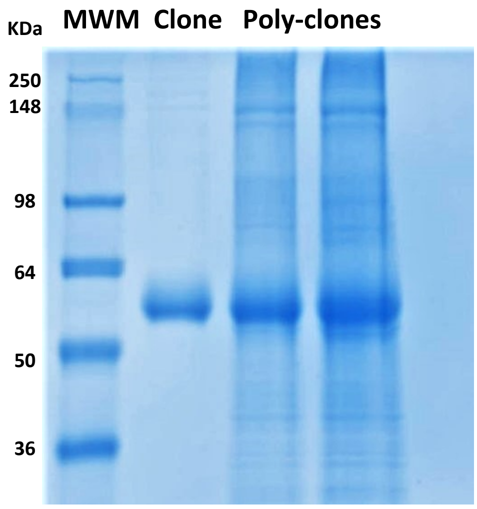

Supplementary Figure 11. Unpurified cell culture supernatants. Comparison of antigen/total protein ratio between polyclones and selected clone. SDS-PAGE, Coomassie Blue staining. MWM: Protein Molecular weight marker. Representative and qualitative figure obtained during clone screening and clone isolation by the end point dilution method. Uncropped and unprocessed scans of this image is provided in the Source Data file.

Supplementary Figure 12:

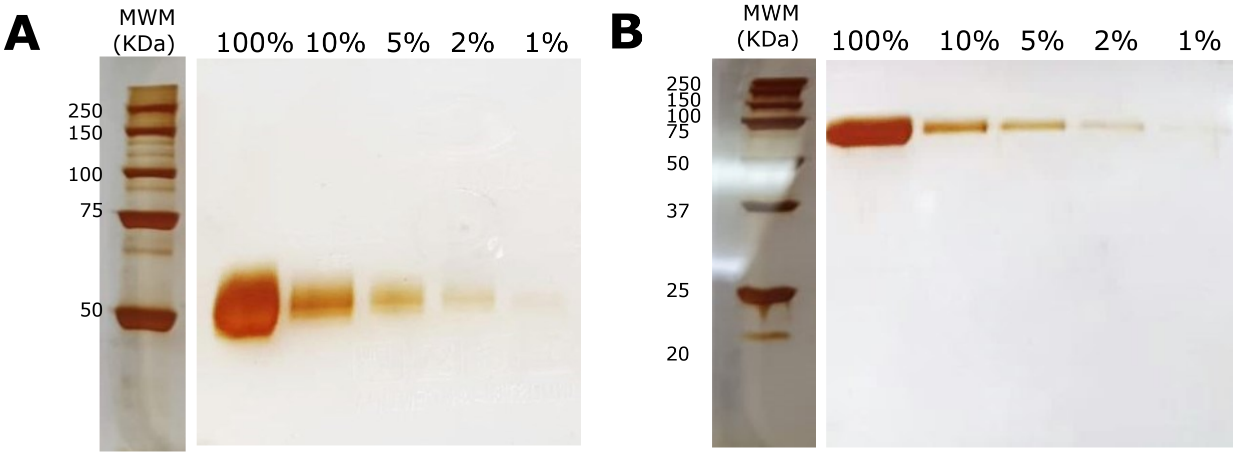

Supplementary Figure 12. Purity of the RBD Gamma antigen. SDS-PAGE, Silver stain. Each lane indicates the relative percentage of protein loads to estimate purity. **A.** Non-reducing conditions (to determine higher molecular weight aggregates); **B.** Reducing conditions (to determine impurities of lower molecular weight). More than three batches of antigen have been prepared with similar results. These results are representative of the purity of a final intermediate of the process, obtained during the development of the Downstream process. All batches of RBD Gamma antigen produced up to the date of this publication (N=5) have been released with a purity greater than 95% using, among other purity methods, SDS-PAGE stained with silver or sensitive Coomassie. Uncropped and unprocessed scans of this image is provided in the Source Data file.

Supplementary tables

Supplementary Table 1. Local and systemic adverse events: Incidence, frequency and severity by vaccine group and doses.								
**Dose**	**ARVAC CG 25-µg Group A**				**ARVAC CG 50-µg Group B**			
**Administration**	**First or second ($N = 60$)**	**First ($N = 60$)**	**Second ($N = 59$)**	P^A	**First or second ($N = 20$)**	**First ($N = 20$)**	**Second ($N = 18$)**	P^A
**Participants with at least one local adverse event, incidence (frequency)**								
**Any grade**	46 (76.7)	41 (68.3)	28(47.5)	*0.026*	12 (60.0)	12 (60.0)	5 (27.8)	*ns*
P^B					*ns*	*ns*	*ns*	
**Grade 1**	45 (75.0)	41 (68.3)	28 (47.5)	*0.026*	12 (60.0)	12 (60.0)	4 (22.2)	*0.025*
P^B					*ns*	*ns*	*ns*	
**Grade 2**	7 (11.7)	5 (8.3)	2 (3.4)	*ns*	2 (10)	1 (5.0)	1 (5.6)	*ns*
P^B					*ns*	*ns*	*ns*	
**Local adverse events overall frequency, N (%) [C]**								
**Any**	129 (100)	71 (100)	58 (100)		34 (100)	25 (100)	9 (100)	
**Grade 1**	117 (90.7)	62 (87.3)	55 (94.8)		31 (91.2)	24 (96)	7 (77.8)	
**Grade 2**	12 (9.3)	9 (12.7)	3 (5.2)		3 (8.8)	1 (4)	2 (22.2)	
**Grade 3**	0 (0)	0 (0)	0 (0)		0 (0)	0 (0)	0 (0)	
**Grade 4**	0 (0)	0 (0)	0 (0)		0 (0)	0 (0)	0 (0)	
**Participants with at least one systemic adverse event, N (%)**								
**Any Grade**	28 (46.7)	20 (33.3)	21 (35.6)	ns	8 (40.0)	8 (40.0)	4 (22.2)	*ns*
P^B					*ns*	*ns*	*ns*	
**Grade 1**	28 (46.7)	19 (31.7)	21 (35.6)	ns	8 (40)	7 (35)	4 (22.2)	*ns*
P^B					*ns*	*ns*	*ns*	
**Grade 2**	6 (10)	4 (6.7)	4 (6.8)	ns	1 (5)	1 (5)	0 (0)	*ns*
P^B					*ns*	*ns*	*ns*	
**Systemic adverse events overall frequency. N (%) [C]**								
**Any**	101 (100)	53 (100)	48 (100)		37 (100)	25 (100)	12 (100)	
**Grade 1**	90 (89.1)	48 (90.6)	42 (87.5)		34 (91.9)	22 (88)	12 (100)	
**Grade 2**	11 (10.9)	5 (9.4)	6 (12.5)		3 (8.1)	3 (12)	0 (0)	
**Grade 3**	0 (0)	0 (0)	0 (0)		0 (0)	0 (0)	0 (0)	
**Grade 4**	0 (0)	0 (0)	0 (0)		0 (0)	0 (0)	0 (0)	

Data are expressed as number of individuals, N (percentage, %) or median (interquartile range, IQR).
[A] Exact *P* Value. First vs. Second dose. Two-sided Fisher's exact test. ns: $P>0.05$.
[B] Exact *P* value. 25-µg vs 50-µg. Two-sided Fisher's exact test. ns: $P>0.05$.
[C] Overall adverse events in the same study group.

Supplementary Table 2: Neutralizing antibody and seroconversion analysis against Ancestral SARS-CoV-2 (Wuhan), Gamma, Delta, Omicron BA.1 and Omicron BA.5 after 25 or 50 µg of ARVAC CG as Booster Dose in Participants.

**SARS-CoV-2 variant**	**Ancestral (Wuhan)**		**Gamma**		**Delta**		**Omicron BA.1**		**Omicron BA.5**	
**ARVAC CG Dose**	**25 µg**	**50 µg**	**25 µg**	**50 µg**	**25 µg**	**50 µg**	**25 µg**	**50 µg**	**25 µg**	**50 µg**
No. of participants evaluated[a]	(N=60)	(N=20)	(N=60)	(N=20)	(N=60)	(N=20)	(N=60)	(N=20)	(N=60)	(N=20)
**Before booster**										
**GMT[b]**	**67.81**	**25.99**	**50.8**	**24.25**	**38.05**	**21.11**	**32.75**	**23.43**	**8.574**	**7.21**
(95% CI)[c]	(46.88-98.07)	(13.46-50.2)	(34.6-74.58)	(12.32-47.74)	(26.57-54.49)	(10.81-41.22)	(23.63-45.38)	(11.26-48.75)	(6.895-10.66)	(4.778-10.88)
**Day 14**										
**GMT[b]**	**851.2**	**776**	**841.4**	**749.6**	**430.5**	**388**	**420.7**	**699.4**	**73.52**	**93.7**
(95% CI)[c]	(569.3-1273.0)	(370.4-1626.0)	(568.8-1245.0)	(397.0-1415.0)	(288.4-642.6)	(200.4-751.2)	(273.3-647.5)	(391.2-1250.0)	(52.0-104.0)	(46.4-189.1)
**GMFR[d]**	**12.6**	**29.9**	**16.6**	**30.9**	**11.3**	**18.4**	**12.8**	**29.9**	**8.6**	**13.0**
(95% CI)[c]	(8.8-17.9)	(12.6-70.6)	(11.8-23.4)	(13.4-71.5)	(7.8-16.5)	(8.2-41.1)	(9.2-18.0)	(13.0-68.3)	(6.1-12.0)	(6.0-28.4)
Two-sided Mann Whitney test *P*	0.0448		ns		ns		0.0297		ns	
**4× Seroconversion at day 14[e]**										
**Percentage of participants (95% CI)**	**88.3** (77.8-94.2)	**90.0** (64.0-94.8)	**90.0** (79.9-95.3)	**85.0** (64.0-94.8)	**80.0** (68.2-88.2)	**85.0** (64.0-94.8)	**93.3** (84.1-97.4)	**85.0** (64.0-94.8)	**80.0** (68.2-88.2)	**80.0** (58.4-91.9)
Two-sided Fisher's exact test, *P*	ns		ns		ns		ns		ns	
Two-sided Chi-square test, *P*	ns		ns		ns		ns		ns	
**10× Seroconversion at day 14[f]**										
**Percentage of participants (95% CI)**	**45.0** (33.1-57.5)	**70.0** (48.1-85.5)	**61.7** (49.0-72.9)	**70.0** (48.1-85.5)	**40.0** (28.6-52.6)	**65.0** (43.3-81.9)	**48.3** (36.2-60.7)	**75.0** (53.1-88.8)	**38.3** (27.1-51.0)	**55.0** (34.2-74.2)
Fisher's exact test, *P*	ns		ns		ns		0.0427		ns	
Chi-square test, *P*	ns		ns		ns		0.0379		ns	

[a] The number of participants with non-missing data at baseline or at 28 days in shown.

[b] GMT: Geometric Mean titer of nAb against the specified virus variant. Antibody values assessed by means of live virus neutralizing antibody assay that were reported as being below the lower limit of detection (LLOD; 8 for Ancestral SARS-CoV-2, Gamma, Delta, Omicron BA.1 and Omicron BA.5) were replaced by 0.5 times the LLOD.

[c] The 95% confidence intervals were calculated on the basis of the t-distribution of log-transformed values or difference in the log-transformed values for geometric mean titer and factor change in geometric mean titer, respectively, then back-transformed to the original scale

[d] GMFR: Fold change in the geometric mean titer respect to before booster antibody titers.

[e] 4× Seroconversion was defined as a change from below the LLOD to at least 4 times the LLOD, or an increase by a factor of at least four if the baseline value was greater than or equal to the LLOD; the comparison was with the baseline value. Percentages were based on the number of participants with non-missing data at baseline and the corresponding time point; 95% confidence intervals were calculated with the use of the Wilson/Brown method.

[f] 10× Seroconversion was defined as a change from below the LLOD to at least 10 times the LLOQ, or an increase by a factor of at least ten if the baseline value was greater than or equal to the LLOQ; the comparison was with the baseline value. Percentages were based on the number of participants with non-missing data at baseline and the corresponding time point; 95% confidence intervals were calculated with the use of the Wilson/Brown method.

ns: $P>0.05$

Supplementary Table 3: Neutralizing antibody and seroconversion analysis against Ancestral SARS-CoV-2 (Wuhan), Gamma, Delta, Omicron BA.1 and Omicron BA.5 after 25 or 50 µg of ARVAC CG as Booster Dose in Participants with BBIBP-CorV primary vaccination scheme.

**SARS-CoV-2 variant**	**Ancestral (Wuhan)**		**Gamma**		**Delta**		**Omicron BA.1**		**Omicron BA.5**	
**ARVAC CG Dose**	**25 µg**	**50 µg**	**25 µg**	**50 µg**	**25 µg**	**50 µg**	**25 µg**	**50 µg**	**25 µg**	**50 µg**
No. of participants evaluated[a]	(N=20)	(N=14)	(N=20)	(N=14)	(N=20)	(N=14)	(N=20)	(N=14)	(N=20)	(N=14)
**Before booster**										
**GMT[b]**	**33.1**	**15.2**	**29.9**	**16.0**	**17.8**	**13.8**	**19.7**	**15.2**	**7.5**	**5.9**
(95% CI)[c]	(16.4-66.8)	(8.7-26.5)	(14.8-60.4)	(7.4-34.5)	(9.5-33.2)	(7.3-25.9)	(10.3-37.7)	(6.3-36.7)	(4.9-11.4)	(4.2-8.4)
**Day 14**										
**GMT[b]** (95% CI)[c]	**477.7** (210.1-1086.0)	**927.5** (353.0-2436.0)	**512** (245.1-1070.0)	**974.5** (434.6-2185.0)	**294.1** (129.6-667.2)	**463.7** (212.0-1014.0)	**247.3** (100.9-605.8)	**760.8** (354.2-1634.0)	**48.5** (25.1-93.9)	**110.3** (44.7-272.5)
**GMFR[d]** (95% CI)[c]	**14.4** (7.1-29.3)	**60.9** (22.7-163.3)	**25.3** (8.7-33.9)	**60.9** (23.9-155.1)	**16.6** (8.2-33.4)	**33.6** (13.9-81.1)	**21.7** (6.3-25.1)	**50.0** (18.4-135.9)	**15.7** (3.5-12.0)	**18.6** (7.1-48.3)
Two-sided Mann Whitney test *P*	0.0154		0.0118		ns		0.0160		0.0459	
**4× Seroconversion at day 14[e]**										
**Percentage of participants (95% CI)**	**85.0** (64.0-94.8)	**92.9** (68.5-99.6)	**85.0** (64.0-94.8)	**92.9** (68.5-99.6)	**85.0** (64.0-94.8)	**92.9** (68.5-99.6)	**95.0** (76.4-99.7)	**92.9** (68.5-99.6)	**70.0** (48.1-85.5)	**85.7** (60.1-97.5)
Two-sided Fisher's exact test. *P*	ns		ns		ns		ns		ns	
Two-sided Chi-square test. *P*	ns		ns		ns		ns		ns	
**10× Seroconversion at day 14[f]**										
**Percentage of participants (95% CI)**	**50.0** (29.9-70.1)	**92.9** (68.5-99.6)	**70.0** (48.1-85.5)	**92.9** (68.5-99.6)	**50.0** (29.9-70.1)	**78.6** (52.4-92.4)	**45.0** (25.8-65.8)	**85.7** (60.1-97.5)	**30.0** (14.5-51.9)	**64.3** (38.8-83.7)
Two sided Fisher's exact test. *P*	0.0110		ns		ns		0.0302		ns	
Two sided Chi-square test. *P*	0.0086		ns		ns		0.0162		0.0475	

[a] The number of participants with non-missing data at baseline or at 28 days in shown.

[b] GMT: Geometric Mean titer of nAb against the specified virus variant. Antibody values assessed by means of live virus neutralizing antibody assay that were reported as being below the lower limit of detection (LLOD; 8 for Ancestral SARS-CoV-2. Gamma. Delta. Omicron BA.1 and Omicron BA.5) were replaced by 0.5 times the LLOD.

[c] The 95% confidence intervals were calculated on the basis of the t-distribution of log-transformed values or difference in the log-transformed values for geometric mean titer and factor change in geometric mean titer. respectively. then back-transformed to the original scale

[d] GMFR: Fold change in the geometric mean titer respect to before booster antibody titers.

[e] 4× Seroconversion was defined as a change from below the LLOD to at least 4 times the LLOD. or an increase by a factor of at least four if the baseline value was greater than or equal to the LLOD; the comparison was with the baseline value. Percentages were based on

the number of participants with non-missing data at baseline and the corresponding time point; 95% confidence intervals were calculated with the use of the Wilson/Brown method.

[f] 10× Seroconversion was defined as a change from below the LLOD to at least 10 times the LLOQ. or an increase by a factor of at least ten if the baseline value was greater than or equal to the LLOQ; the comparison was with the baseline value. Percentages were based on the number of participants with non-missing data at baseline and the corresponding time point; 95% confidence intervals were calculated with the use of the Wilson/Brown method.

ns: P>0.05

Supplementary Table 4. Comparison of the nAb response in male or female individuals after a booster dose of ARVAC CG.

Variant	Dose	Sex[a]	N[b]	nAb Titer GMT (CI 95%)[c]						4× seroconversion[d]		
				d1		d14		P^e	P^f	%	95% CI	P^g
Ancestral	25 µg	M	29	**65.6**	(36.7-116.9)	**682.1**	(392.5-1185	0.0003	ns	**90.3**	75.1 - 96.7	ns
		F	31	**70.0**	(42.5-115.3)	**1047**	(572.7-1915	0.0001		**86.2**	69.4 - 94.5	
	50 µg	M	8	**26.9**	(7.3-99.2)	**861.1**	(262.1-2829	0.0004	ns	**83.3**	55.2 - 97.0	ns
		F	12	**25.4**	(10.7-60.5)	**724.1**	(240.3-2182	0.0002		**100.0**	67.6 - 100.0	
Gamma	25 µg	M	29	**45.8**	(27.1-77.5)	**750.5**	(422.3-1334	0.0001	ns	**90.3**	75.1 - 96.7	ns
		F	31	**56.0**	(31.2-100.5)	**936.4**	(532.9-1645	0.0001		**89.7**	73.6 - 96.4	
	50 µg	M	8	**19.0**	(5.4-67.6)	**664.0**	(238.4-1849	0.0007	ns	**83.3**	55.2 - 97.0	ns
		F	12	**28.5**	(11.4-71.3)	**812.7**	(315.8-2091)	0.0001		**87.5**	52.9 - 99.4	
Delta	25 µg	M	29	**32.8**	(19.7 - 54.6)	**403.1**	(238.7 - 680.8)	0.0001	ns	**93.1**	78.0 - 98.8	0.0141
		F	31	**43.8**	(25.8 - 74.3)	**457.8**	(243.8 - 859.6)	0.0001		**67.7**	50.1 - 81.4	
	50 µg	M	8	**19.0**	(5.8 - 62.5)	**394.8**	(135.4 – 1151)	0.0004	ns	**87.5**	52.9 - 99.4	ns
		F	12	**22.6**	(8.8 - 58.4)	**383.6**	(143.4 – 1026)	0.0005		**83.3**	55.2 - 97.0	
Omicron BA.1	25 µg	M	29	**30.5**	(19.0-48.9)	**366.4**	(193.1-695.2)	0.0001	ns	**93.5**	79.3 - 98.9	ns
		F	31	**35.0**	(21.7-56.4)	**478.8**	(259.2-884.3)	0.0001		**93.1**	78.0 - 98.8	
	50 µg	M	8	**19.0**	(6.0-60.0)	**430.1**	(148.9-1245)	0.022	ns	**83.3**	55.2 - 97.0	ns
		F	12	**26.9**	(9.0-80.6)	**966.5**	(461.9-2022)	0.0001		**87.5**	52.9 - 99.4	
Omicron BA.5	25 µg	M	29	**8.8**	(6.3 - 12.3)	**62.5**	(39.3 - 99.5)	0.0001	ns	**79.3**	61.6 - 90.2	ns
		F	31	**8.4**	(6.2 - 11.3)	**85.6**	(50.3 - 145.8)	0.0001		**80.6**	63.7 - 90.8	
	50 µg	M	8	**6.2**	(4.0 - 9.5)	**58.7**	(19.7 - 175.0)	0.0004	ns	**75.0**	40.9 - 95.6	ns
		F	12	**8.0**	(4.1 - 15.8)	**128.0**	(46.6 - 351.9)	0.0015		**83.3**	55.2 - 97.0	
**Variant**	**Dose**	**Sex**	**N**	**d1**		**d28**		P^d	P^b	**%**	**95% CI**	P^c
Ancestral	25 µg	M	29	**65.6**	(36.7-116.9)	**698.6**	(425.3-1147)	0.0001	ns	**76.7**	59.1 - 88.2	ns
		F	30	**67.0**	(40.3-(111.1)	**601.9**	(344.1-1053)	0.003		**89.7**	73.6 - 96.4	
	50 µg	M	7	**26.3**	(5.5-124.6)	**512.0**	(206.8-1268)	0.0034	ns	**81.8**	52.3 - 96.8	ns
		F	11	**28.2**	(11.2-71.29)	**329.4**	(120.6-899.5)	0.002		**85.7**	48.7 - 99.3	
Gamma	25 µg	M	29	**45.8**	(27.1-77.46)	**443.6**	(269-731.5)	0.0001	ns	**70.0**	52.1 - 83.3	ns
		F	30	**54.4**	(29.8-99.49)	**456.1**	(276.7-751.9)	0.0001		**82.8**	65.5 - 92.4	
	50 µg	M	7	**17.7**	(3.9-79.18)	**344.6**	(130.7-908.2)	0.003	ns	**81.8**	52.3 - 96.8	ns
		F	11	**32.0**	(12.1-84.99)	**309.3**	(127.5-749.9)	0.0033		**85.7**	48.7 - 99.3	
Omicron	25 µg	M	29	**68.8**	(39.6-119.4)	**288.5**	(147.3-565.2)	0.0002	0.8776	**51.5**	35.2 - 67.5	0.908
		F	30	**67.0**	(40.3-111.4)	**308.0**	(178.1-532.6)	0.0001		**50.0**	32.1 - 67.9	
	50 µg	M	7	**23.8**	(5.7-99.02)	**231.9**	(97.9-549.2)	0.0022	0.2993	**81.8**	52.3 - 96.8	0.6052
		F	11	**30.5**	(9.2-98.41)	**423.8**	(179.2-1002)	0.002		**71.4**	35.9 - 94.9	

[a] Sex. M, male; F: female.
[b] Number of participants with non-missing data at the time point (or at baseline).
[c] GMT: Geometric Mean titer of nAb against the specified virus variant. Antibody values assessed by means of live virus neutralizing antibody assay that were reported as being below the lower limit of detection (LLOD; 8 for Ancestral SARS-CoV-2, Gamma, Delta, Omicron BA.1 and Omicron BA.5) were replaced by 0.5 times the LLOD. The 95% confidence intervals were calculated on the basis of the t-distribution of log-transformed values or difference in the log-transformed values for geometric mean titer and factor change in geometric mean titer, respectively, then back-transformed to the original scale.
[d] 4× Seroconversion was defined as a change from below the LLOD to at least 4 times the LLOD, or an increase by a factor of at least four if the baseline value was greater than or equal to the LLOD; the comparison was with the pre-vaccination baseline value. Percentages were based on the number of participants with non-missing data at baseline and the corresponding time point; 95% confidence intervals were calculated with the use of the Wilson/Brown method.
[e] Exact *P* Value. d1 vs. d14 or d28, respectively. Two-tailed Wilcoxon pair-matched test. ns: *P*>0.05.
[f] Exact *P* value. D14 or d28 M vs d12 or d28 F. Two-sided Mann Whitney test t. ns: *P*>0.05.
[g] Exact *P* value. M vs F. Two-sided Fisher's exact test. ns: *P*>0.05.

Supplementary Table 5: Neutralizing antibody and seroconversion Analysis against Ancestral SARS-CoV-2 (Wuhan), Gamma, and Omicron BA.1 after 28 days of administration of 25 or 50 µg of ARVAC CG as Booster Dose in Participants.

**SARS-CoV-2 variant**	**Ancestral**		**Gamma**		**Omicron BA.1**	
**ARVAC CG Dose**	**25 µg**	**50 µg**	**25 µg**	**50 µg**	**25 µg**	**50 µg**
No. of participants evaluated[a]	(N=58)	(N=18)	(N=58)	(N=18)	(N=58)	(N=18)
**Before booster**						
GMT[b]	**69.6**	**27.4**	**52.2**	**25.4**	**33.6**	**23.5**
(95% CI)[c]	(48.25-100.4)	(13.3-56.6)	(35.5-76.86)	(12.0-53.6)	(24.16-46.64)	(10.5-52.5)
**Day 28**						
GMT[b]	**658.1**	**391.0**	**443.6**	**322.5**	**299.0**	**335.2**
(95% CI)[c]	(455.1-951.5)	(204.3-748.3)	(313.3-628.1)	(178.6-582.5)	(195.2-458.1)	(187.5-599.2)
GMFR[d]	**9.5**	**14.3**	**8.5**	**12.7**	**8.9**	**14.3**
(95% CI)[c]	(6.5-13.1)	(5.7-35.4)	(5.8-12.4)	(5.1-31.5)	(6.1-13.0)	(5.9-34.3)
Mann Whitney test. *P*	ns		ns		ns	
**4× Seroconversion at day 28[e]**						
**Percentage of participants**	**82.8**	**83.3**	**75.9**	**83.3**	**75.9**	**83.3**
**(95% CI)**	(71.1-90.4)	(60.8-94.2)	(63.5-85.0)	(60.8-94.2)	(63.5-85.0)	(60.8-94.2)
Fisher's exact test, *P*	ns		ns		ns	
Chi-square test, *P*	ns		ns		ns	
**10× Seroconversion at day 28[f]**						
**Percentage of participants**	**39.7**	**72.2**	**43.1**	**61.1**	**37.9**	**61.1**
**(95% CI)**	(28.1-52.5)	(49.1-87.5)	(31.2-55.9)	(38.6-79.7)	(26.6-50.8)	(38.6-79.7)
Fisher's exact test, *P*	0.0289		ns		ns	
Chi-square test, *P*	0.0156		ns		ns	

[a] Shown is the number of participants with non-missing data at the time point (or at baseline).

[b] Antibody values assessed by means of live virus neutralizing antibody assay that were reported as being below the lower limit of detection (LLOD; 8 for Ancestral SARS-CoV-2, Gamma, Delta, Omicron BA.1 and Omicron BA.5) were replaced by 0.5 times the LLOD.

[c] The 95% confidence intervals were calculated on the basis of the t-distribution of log-transformed values or difference in the log-transformed values for geometric mean titer and factor change in geometric mean titer (GMT), respectively, then back-transformed to the original scale.

[d] GMFR: Fold change in the GMT titer respect to baseline antibody titers.

[e] 4× Seroconversion was defined as a change from below the LLOD to at least 4 times the LLOD, or an increase by a factor of at least four if the baseline value was greater than or equal to the LLOD; the comparison was with the baseline value. Percentages were based on the number of participants with no missing data at baseline and the corresponding time point; 95% confidence intervals were calculated with the use of the Wilson/Brown method.

[f] 10× Seroconversion was defined as a change from below the LLOD to at least 10 times the LLOD, or an increase by a factor of at least ten if the baseline value was greater than or equal to the LLOD; the comparison was with the baseline value. Percentages were based on the number of participants with no missing data at baseline and the corresponding time point; 95% confidence intervals were calculated with the use of the Wilson/Brown method.

ns: $P>0.05$.

Supplementary Methods

1- Vaccine manufacture, composition, and quality control.

ARVAC Quality Summary

ARVAC is a recombinant adjuvanted protein subunit vaccine candidate for intramuscular administration. Clinical lot used for this study was packaged in single dose pharmacopeial type I glass vials with rubber stoppers and aluminum seals.

Formulation and manufacturer

Each 0.5-mL dose of the vaccine candidate contains 25 μg or 50 μg of antigen, adsorbed on 0.5 mg of aluminum hydroxide, in a vehicle composed of dibasic sodium phosphate, monobasic sodium phosphate, L-histidine, sodium chloride, mannitol, and water for injection.

Both the antigen (active pharmaceutical ingredient) and the vaccine candidate (finished product) are manufactured under GMP in plants of Laboratorio Pablo Cassará S.R.L located in Buenos Aires, Argentina.

Cell substrate

The cell substrate is a high-productivity clone generated from a CHO-S (Chinese Hamster Ovary) cell line, cultured in high-density suspension, using an animal component-free medium.

Genetic construction and expression of the recombinant antigen

The gene of interest contains the amino acids R319 to K537 corresponding to the receptor binding domain of the Spike protein, of the Variant of Concern (VOC) Gamma (includes the relevant mutations: K417T, E484K and N501Y).

The synthetic DNA cassette (GenScript Biotech Corporation) containing the open reading frame (ORF) was introduced in a proprietary plasmid vector for protein expression in mammalian cells. Expression is regulated under a Cytomegalovirus (CMV) promoter. The synthetic cassette possesses the codon adaptation for expression in CHO cells. The genetic construct uses the signal peptide of the SARS CoV-2 Spike protein (amino acids M1 to Q14), so that the recombinant antigen can be processed and exported to the extracellular space.

The genetic material was introduced into the host cell by lipofection, and highly productive stable clones were isolated by end point dilution and screened for productivity using ELISA (Enzyme-Linked Immuno-Sorbent Assay) and SDS-PAGE (sodium dodecyl sulfate polyacrylamide gel electrophoresis). Antigen identity was verified by SDS-PAGE Western Blot, and by functional ELISA for human ACE2 receptor affinity. The expected aminoacidic sequence was confirmed by Liquid Chromatography Electrospray Ionization Tandem Mass Spectrometric (LC/ESI-MS/MS) studies (Protagen, Germany).

The nucleotide sequence (coding region) of the expression vector and of the specific messenger RNA (through complementary DNA, cDNA) were confirmed in the selected clone. Functionality of the signal peptide and productivity of the selected clone is illustrated in Supplementary Fig. 11, showing the results of an SDS PAGE

run of unpurified culture supernatants comparing this clone against polyclones. The selected clone shows a much higher antigen / total protein ratio.

Master and working cell banks were prepared from the selected clone and controlled following the recommendations of the ICH guidelines (International Council for Harmonization of Technical Requirements for Pharmaceuticals for Human Use) to establish their identity, purity, and stability.

Antigen manufacturing process

Upstream

The upstream process is carried out in a single-use perfusion bioreactor (SUB).

Perfusion allows the renewal of the culture medium through a cell retention/separation system (in our case it is carried out by an alternate tangential flow filtration device). Conditioned cell-free medium containing the exported active ingredient is harvested and replaced by fresh medium, maintaining high viability and cell density for ca. 30 days. After a first growth phase, daily harvested material is filtered, controlled, and used as starting material for the downstream process.

Downstream

The antigen is soluble and constitutes ca. 50% of the total proteins in the harvest.

The downstream process consists of the following purification steps:

- Concentration and change of harvest buffer.

- Capture chromatography to reduce water content and residual DNA and host cell proteins.

- Specific ion exchange chromatography to eliminate antigen-related impurities (aggregates, misfolded protein) and further reduce residual DNA and host cell proteins.

- Ion exchange chromatography polishing step to reach target purity of the antigen.

- Diafiltration and final filtration.

Between each chromatographic step, tangential diafiltration is used to change the buffers and adjust pH and ionic strength of intermediates for the next step.

The purified antigen solution contains similar soluble excipients of the vaccine candidate and is stored at -20°C. Supplementary Fig. 12 shows the high purity of the antigen in a silver stained SDS PAGE run of the purified antigen.

Vaccine candidate (finished product) manufacturing process

Soluble excipients are dissolved in water for injection and the solution is sterilized by filtration. Sterile aluminum hydroxide is dispersed in the filtered solution and the antigen is added under aseptic conditions and the suspension is stirred in a closed vessel to accelerate adsorption. The suspension is filled into the final

containers and sealed under aseptic conditions. The clinical lot used in this study was filled into single-dose vials. Stability batches filled into multidose vials and ampoules are currently being studied for stability according to ICH guidelines.

Quality Control

Each clinical lot of antigen is released after being tested for: appearance (visual inspection), pH (potentiometry); identity (ELISA, ACE2 receptor affinity, molecular weight), antigen-related impurities (reducing and non-reducing SDS, RP-HPLC and SEC), process – related impurities (host cell proteins by ELISA and residual DNA by real time PCR); protein concentration (UV spectrophotometry), EC50 (ACE2 receptor binding functional ELISA), microbial count and bacterial endotoxins (endpoint chromogenic LAL).

Each clinical lot of vaccine candidate is released after being tested for: appearance (visual inspection), resuspendability (visual inspection), pH (potentiometry); identity (ELISA, ACE2 receptor affinity), extractable volume (gravimetry); aluminum content (atomic absorption); free antigen (non-reducing PAGE); EC50 (functional ACE2 receptor binding ELISA); sterility; bacterial endotoxins (endpoint chromogenic LAL) and safety (in vivo biological reactivity).

Specifications have been established for each parameter based on development data and pharmacopeial standards used for other recombinant protein vaccines.

Stability

Antigen is stable for at least 15 months at -20°C and 1 week at 2 – 8 °C. It remains stable after at least three freeze - thaw cycles.

Vaccine candidate is stable for at least 15 months at 2 – 8 °C and up to at least 1 week at 25 ± 2°C in single-dose vials. It should not be frozen.

2- Determination of SARS-CoV-2 Nucleoprotein (N)-specific antibody levels in serum.

Nucleoprotein (N)-specific antibody responses (IgG) were evaluated by indirect ELISA. Recombinant N protein (0.25 µg/well) was used to coat plates and HRP conjugated anti-human IgG was used to detect Ab (Jackson ImmunoResearch Laboratories Ink, Code: 109135-088, Lot number:135723, final dilution 1:8000). Results were read at 450 nm in an 800TS microplate reader with Gen5 software v3.10.06 (Biotek Instrument Ink.) to collect endpoint ELISA data. End-point cut-off values for serum titer determination were calculated as the mean specific optical density (OD) plus 3 standard deviations (SD) from pre-pandemic sera of healthy donors diluted 1:200 in assay diluent. Titers were established as the reciprocal of the last dilution yielding an OD higher than the cut-off.

Study synopsis

**Protocol N°:**	ARVAC-F1-001
**Sponsor:**	Laboratorio Pablo Cassará<br>Carhué 1096<br>Ciudad Autónoma de Buenos Aires
**Official study title:**	Phase 1 study to evaluate safety, tolerability and immunogenicity of a new recombinant protein-based vaccine against SARS-CoV-2 (ARVAC CG), in a population of healthy adult volunteers previously vaccinated against SARS-CoV-2 virus.
**Version N° and date:**	V. 3.0 – February 25, 2022
**Clinical pharmacology phase:**	Phase I
**Type of study:**	Open-label, prospective, phase 1 study.
**Primary objective:**	To describe the safety and tolerability profile of ARVAC CG vaccine (recombinant protein vaccine against SARS-CoV-2) in a population of healthy adult volunteers, previously vaccinated against SARS-CoV-2 virus.
**Secondary objectives:**	- To compare the safety profile depending on the primary vaccination schedule received.<br>- To compare the safety profile between volunteers who receive a 2-dose vaccination of 25 µg of antigen and those who receive a 2-dose vaccination of 50 µg of antigen.<br>- To Describe the immune response triggered by the application of ARVAC CG vaccine, after each of the vaccine doses under study, and depending on the different variables involved.
**Population:**	A total of 80 healthy adult volunteers meeting all of the eligibility criteria named below will be included.
**Vaccine under study:**	Vaccine against SARS-CoV-2 virus based on a recombinant protein antigen containing the human ACE2 Receptor Binding Domain region of SARS-CoV-2 Spike protein, adjuvanted with aluminum hydroxide.
**Inclusion criteria:**	1. Male or female participants between 18 and 55 years of age.<br>2. With the ability and willingness to comply with the prohibitions and restrictions specified in the protocol.<br>3. Healthy volunteers, which will be determined by the history referred to interrogation, physical examination and principal investigator's criteria.<br>4. In fertile female volunteers, negative pregnancy test at the beginning of the study and commitment to use a contraceptive method from the date of signing the consent form until 3 months after vaccine study application. Use of an hormonal

	contraceptive method must begin at least 28 days prior to study vaccine application. The investigator should assess potential contraceptive method failure (e.g. non-compliance, recent onset) in relation to vaccination. Acceptable effective methods for this study include:<br><br>a) hormonal contraceptive method:<br><br>i) combined (containing estrogen and progestin) associated with the inhibition of ovulation (oral, intravaginal or transdermal);<br><br>ii) with progestin only, associated with the inhibition of ovulation (oral, injectable or implantable);<br><br>b) intrauterine device;<br><br>c) intrauterine hormone release system;<br><br>d) bilateral tubal ligation/occlusion procedure;<br><br>e) single couple with vasectomy;<br><br>f) sexual abstinence, which will be considered effective only if it is defined as abstaining from heterosexual relations from the date of signing the consent until 3 months after receiving the study vaccine. The reliability of sexual abstinence should be assessed in relation to the duration of the study and the participant's usual and preferred lifestyle.<br><br>5. Participant who agrees to do not donate bone marrow, blood or blood products until 3 months after the last dose of study vaccine;<br><br>6. Must be able to read, understand, and complete electronic questionnaires about signs and symptoms of COVID-19 surveillance;<br><br>7. Negative PCR for the SARS-CoV-2 virus.<br><br>8. With laboratory analysis without clinically significant variations within the 30 days prior to receiving the first dose of the study vaccine, which must include:<br><br>a) complete blood count (hemoglobin (Hb), leukocyte count and leukocyte formula, platelet count;<br><br>b) complete liver test: total and direct bilirubin, transaminases (alanine aminotransferase [ALT or GPT] and aspartate aminotransferase [AST or GOT]), lactate dehydrogenase (LDH), alkaline phosphatase (ALF).<br><br>c) biochemistry: glycemia, urea, creatinine;<br><br>d) Qualitative C-reactive protein (PCR);<br><br>e) Complete urine.<br><br>9. Capable of granting their informed consent signed and dated by the volunteer under study, and the authorized physician.

Exclusion criteria:	1. History of SARS-CoV-2 infection or known previous disease, within 60 days prior to study entry (at least 60 days from epidemiological discharge).<br><br>2. Administration of any other commercial vaccine or not based on:<br><br>a. Live attenuated virus within 28 days prior to study entry.<br><br>b. Killed virus within 14 days prior to study entry.<br><br>3. Individuals that have not received a complete primary vaccination schedule against SARS-CoV-2 virus (1 or 2 doses, depending on the vaccine used in the primary schedule).<br><br>4. Administration of complete primary vaccination schedule against SARS-CoV-2 virus (1 or 2 doses, depending on the vaccine received), within 4 months prior to the start of the study.<br><br>5. Administration of an additional or booster dose after a complete primary vaccination schedule against SARS-CoV-2 virus.<br><br>6. Individuals that have scheduled to receive any other commercial vaccine in the following 3 months.<br><br>7. Individuals that have participated in a research study within 60 days prior to the start of the study.<br><br>8. Present a history of known allergies or a history of anaphylaxis or any other serious adverse reaction with other vaccines or their excipients.<br><br>9. History of alcoholism or substance abuse that prevents compliance with the characteristics of the protocol.<br><br>10. Acute infectious disease at enrollment (this does not include minor conditions such as diarrhea or mild upper respiratory tract illness) or temperature ≥38. 0°C within 24 hours prior to scheduled study vaccination; entry at a later date is permitted at the discretion of the investigator and after consultation with the Sponsor.<br><br>11. Any laboratory determination alteration with a degree of severity > 1 according to the Common Toxicity Criteria (CTC version 5 – November 2017). Participants with any stable grade 1 abnormality may be considered eligible by the investigator. (grade 1 stable implies a repetition of the sample that persists with an alteration of one grade no greater than 1).<br><br>12. Body Mass Index (BMI) greater than 30 kg/m2 or less than 18 kg/m2.<br><br>13. Individuals currently working in occupations with high exposure to SARS-CoV-2.<br><br>14. History of any clinical condition that affects the function of the immune system, including, but not limited to:<br><br>a. Clinical conditions (e.g. autoimmune disease or possibly immune-mediated disease or known or suspected immunodeficiency; diabetes mellitus type I or II, chronic kidney disease, etc.).

	b. Chronic or recurrent use of systemic corticosteroids in the 6 months prior to study vaccine administration and during the study. A substantially immunosuppressive dose of steroids is considered ≥2 weeks of daily administration of 20 mg prednisone or equivalent.<br><br>c. Administration of antineoplastic and immunomodulatory agents or radiation therapy within 6 months prior to study vaccine administration or during the study.<br><br>15. The volunteer has received an investigational drug (including drugs related to COVID-19 prophylaxis) or used an investigational invasive medical device in the past 30 days, or has received investigational immunoglobulin or monoclonal antibodies within 3 months (participation in an observational study is allowed at the discretion of the investigator, previously informing the Sponsor about this decision).<br><br>16. The participant is pregnant, plans to become pregnant within 3 months after the administration of the vaccine, or is in postpartum or lactation period.<br><br>17. The volunteer has any contraindication to receive intramuscular injections and/or blood draws.<br><br>18. The volunteer has a history of acute polyneuropathy (e.g. Guillan Barré syndrome).<br><br>19. The volunteer underwent a surgical procedure that required hospitalization (defined as hospitalization for more than 24 hours or overnight hospitalization), in the 12 weeks prior to vaccination, or has not recovered completely from surgery that required hospitalization, or is scheduled for surgery that will require hospitalization during the time he/she is expected to participate in the study or within 6 months of study vaccine administration.<br><br>20. Positive serology for hepatitis (HBsAg, Anti-HBc, Anti-HCV).<br><br>21. Positive antibodies against HIV.
**Discontinuation criteria:**	The Investigator could discontinue the participation in the study of any of the participants for any of the following reasons:<br><br>1. Voluntary withdrawal of the volunteer for any reason.<br><br>2. Failure to comply with the administrative requirements of the protocol.<br><br>3. Pregnancy.<br><br>4. Severe allergic reactions.<br><br>5. Any SAE that the participant develops and for which there is no other plausible possible alternative of cause attributable to the event.<br><br>6. Any grade 4 event that the participant develops, both regarding local or systemic reactions, and including fever over 40°C after vaccination, which at the Investigator´s discretion, is related to the vaccine

	under study and there is no other plausible cause that should be considered as cause of the event.<br><br>7. If 2 or more participants present a grade 3 event, of similar characteristics, which, at the Investigator´s discretion, is related to the vaccine under study and there is no other plausible cause that could be considered as the cause of the event.
**Factors to consider:**	**Safety**<br><br>1. Local reactions during the following 7 days after administration of each dose of the vaccine.<br>2. Systemic reactions during the following 7 days after administration of each dose of the vaccine.<br>3. Adverse events (AEs) that occur from the application of the first dose until 1 month after the last dose.<br>4. Serious adverse events (SAEs) that occur from the application of the first dose until 6 months after the last dose.<br>5. Changes in the laboratory results respect from a baseline control, at days 7, 28 and 56 after the firs dose application of the first dose of vaccine. 365 days after the first dose of vaccine, an additional laboratory control will be carried out.<br><br>**Immunogenicity**<br><br>- Determination of neutralizing antibodies (geometric mean titer – GMT) at 14 and 28 days after each vaccine dose (days 0, 14, 28, 42 and 56).<br><br>- Determination of total specific antibodies on day 1 (before the first vaccine dose) and 28 days after each vaccine dose (days 28 and 56).<br><br>- Determination of cellular immune response, specific IFN gamma and IL-4 producing cells directed to RBD (Spike protein region) on day 1 (prior to the first dose) and 28 days after each vaccine dose (day 28 and 56).<br><br>- Neutralizing antibody titer variation respect from baseline after each of the vaccine doses.<br><br>As exploratory variables, antibodies variation in the different volunteers subgroups will be described:<br><br>- Depending on the primary vaccination schedule received.<br><br>- Depending on the vaccine platform used in the primary scheme.<br><br>- Depending on the dose of the study vaccine received (2 doses of 25 µg of antigen or 2 doses of 50 µg of antigen).<br><br>- Depending on the history of having had a previous SARS-CoV-2 infection or not.

	Throughout the study, cases of COVID-19 disease will be recorded, reporting their incidence and the difference between the different groups.
**Study design:**	The study will consist of 7 visits, each of which could last more than 1 day.<br>Visit 1 – Signing of informed consent - Selection of volunteers (Day -30 to -1)<br>Visit 2 – First dose of vaccine (Days 0 to 3).<br>Visit 3 - First face-to-face control (day 7 ± 1).<br>Visit 4 – Second face-to-face control (day 14 ± 2).<br>Visit 5 – Second dose of vaccine (Days 28 to 31 ± 2).<br>Visit 6 - Clinical and immunogenicity control (day 42 ± 3).<br>Visit 7 - Clinical and immunogenicity control (day 56 ± 5).<br>First safety follow-up visit (day 180 ± 15).<br>Second safety and immunogenicity follow-up visit (day 365 ± 20)
**Statistical análisis:**	**Safety analysis**<br>Descriptive statistics for each variable of local, systemic and laboratory reactogenicity reported for each dose and vaccine group.<br>AEs will be classified according to the Medical Dictionary for Terms of Regulatory Activities (MedDRA).<br>Safety analysis will be based on the safety population. Participants will be summarized by group, based on the dose of study vaccine received and based on the type of vaccine received in the primary scheme.<br>Missing data in the self-assessment cards completed by the participants will not be imputed.<br>The comparison of frequencies between groups will be carried out by the chi-square test.<br><br>**Immunogenicity analysis**<br>The statistical analysis of the immunogenicity results will be based primarily on the analysis of the population defined as the immunogenicity population with 1 and 2 doses of vaccine.<br>As previously mentioned, the antibody titer will be analyzed as geometric means calculated from the anti-log of the mean of logs of the antibody titers. Thus, the means and their respective confidence intervals will be obtained.<br>They will also be tabulated based on the dose of study vaccine received, considering previous SARS-CoV-2 infection or not, and based on the vaccine platform received in the primary scheme. The difference between means will be evaluated using the

	Student's t test. A level of 0.05 will be considered statistically significant.
