# Supplementary material for: Safety and immunogenicity of a SARS-CoV-2 Gamma variant RBD-based protein adjuvanted vaccine used as booster in healthy adults
Source: Nat Commun. 2023 Jul 28;14:4551. doi: 10.1038/s41467-023-40272-3 (PMC10382514; doi:10.1038/s41467-023-40272-3)
Supplement: Supplementary file 3 — Reporting Summary [file 41467_2023_40272_MOESM3_ESM.pdf]

nature portfolio

Corresponding author(s): Karina Pasquevich
Juliana Cassataro

Last updated by author(s): Jul 14, 2023

Reporting Summary

Nature Portfolio wishes to improve the reproducibility of the work that we publish. This form provides structure for consistency and transparency in reporting. For further information on Nature Portfolio policies, see our Editorial Policies and the Editorial Policy Checklist.

Statistics

For all statistical analyses, confirm that the following items are present in the figure legend, table legend, main text, or Methods section.

n/a	Confirmed	
☐	☒	The exact sample size (*n*) for each experimental group/condition, given as a discrete number and unit of measurement
☐	☒	A statement on whether measurements were taken from distinct samples or whether the same sample was measured repeatedly
☐	☒	The statistical test(s) used AND whether they are one- or two-sided<br>*Only common tests should be described solely by name; describe more complex techniques in the Methods section.*
☐	☒	A description of all covariates tested
☐	☒	A description of any assumptions or corrections, such as tests of normality and adjustment for multiple comparisons
☐	☒	A full description of the statistical parameters including central tendency (e.g. means) or other basic estimates (e.g. regression coefficient) AND variation (e.g. standard deviation) or associated estimates of uncertainty (e.g. confidence intervals)
☐	☒	For null hypothesis testing, the test statistic (e.g. *F*, *t*, *r*) with confidence intervals, effect sizes, degrees of freedom and *P* value noted<br>*Give P values as exact values whenever suitable.*
☒	☐	For Bayesian analysis, information on the choice of priors and Markov chain Monte Carlo settings
☒	☐	For hierarchical and complex designs, identification of the appropriate level for tests and full reporting of outcomes
☒	☐	Estimates of effect sizes (e.g. Cohen's *d*, Pearson's *r*), indicating how they were calculated

Our web collection on statistics for biologists contains articles on many of the points above.

Software and code

Policy information about availability of computer code

Data collection	Data were collected in a eCRF from Castor EDC. SARS-CoV-2 Spike, RBD and Nucleocapsid IgG were determined by ELISA using a Multiscan Go microplate reader from ThermoScientific with Thermo Scientific SkanIt Software (Spike and RBD) and a 800TS microplate reader (BioTek instrument Ink.) with Gen5 software v3.10.06 (Biotek Instrument Ink.) for anti-N IgG titers.<br>ElisPot Plates were scanned on an ImmunoSpot reader (Cellular Technology Ltd.). Specific spots were counted using the ImmunoSpot 5.0 software.
Data analysis	Statistical analyses were done using GraphPad Prism v8.4.2 (GraphPad Software, San Diego, CA),

For manuscripts utilizing custom algorithms or software that are central to the research but not yet described in published literature, software must be made available to editors and reviewers. We strongly encourage code deposition in a community repository (e.g. GitHub). See the Nature Portfolio guidelines for submitting code & software for further information.

Data

Policy information about availability of data

All manuscripts must include a data availability statement. This statement should provide the following information, where applicable:

- Accession codes, unique identifiers, or web links for publicly available datasets
- A description of any restrictions on data availability
- For clinical datasets or third party data, please ensure that the statement adheres to our policy

All data are presented in the main text or the supplementary materials and are available upon reasonable request from the corresponding author.

Research involving human participants, their data, or biological material

Policy information about studies with human participants or human data. See also policy information about sex, gender (identity/presentation), and sexual orientation and race, ethnicity and racism.

Reporting on sex and gender	In the study, healthy male and female participants with a complete COVID19-primary vaccine scheme (18-55 years-old) were included.<br>Participants sex (male, female) was assigned based on sex assigned at birth, as self-reported by the participant. The numbers of female and male participants are detailed in table 1 and table 2 of the manuscript.<br>In group A, of the 60 participants, 29 participants were male and 31 participants were female.<br>In group B, of the 20 participants, 8 participants were male and 12 participants were female.<br>Of the 18 participants boosted with BNT16b2 from the surveillance strategy implemented by the Ministry of Health of the Province of Buenos Aires, 5 were male and 13 female.<br>Sex-based analysis were performed. Results are presented in supplementary table 4.
Reporting on race, ethnicity, or other socially relevant groupings	No reporting on Race, ethnicity, or other socially relevant groupings have been performed.
Population characteristics	Demographic Characteristics of the Participants in the ARVAC-F1-001 Trial at Enrolment are presented in Table 1 of the manuscript.<br>Demographic Characteristics of the Participants of the surveillance strategy implemented by the Ministry of Health of the Province of Buenos Aires that were used boosted with BNT16b2 at Enrolment are presented in Table 2 of the manuscript.
Recruitment	Recruitment was performed by phone contact with healthy volunteers form a data base and by using mouth to mouth strategy. Limitations of sequential recruitment are discused in the manuscript. No self-selection bias nor other bias that may influence the study results have been detected. The study protocol was initiated on April 10, 2022. Twenty seven out of 107 volunteers (13 males, 14 females) were excluded for not complying with eligibility criteria. Participants were recruited between April 28 and June 23, 2022, and sequentially assigned to one of two vaccine groups, one receiving a 25 µg dose of ARVAC CG (Group A) and the other a 50 µg dose (Group B). A sequential assignment plan was prespecified in the study protocol. In the first stage of enrollment, the first five enrolled participants received the low dose vaccine (25 µg/dose). Only one participant per day was vaccinated. Afterwards in the second stage of enrollment, participants 6th to 10th received the high dose vaccine formulation (50 µg/dose). Only one participant per day was vaccinated. The next fifty-five enrolled participants received the 25 µg/dose (stage 3), and then the last fifteen participants received the 50 µg/dose (stage 4).
Ethics oversight	All participants provided written informed consent before enrolment in the trial and after the nature and possible consequences of the study were explained. The study was conducted according to the Declaration of Helsinki. The trial protocol was approved on March 09, 2022, by the Ethic Committee in Clinical Research of Centro de Estudios Infectológicos S.A., and by the Food and Drugs National Regulatory Agency (Administración Nacional de Medicamentos, Alimentos y Tecnología Médica, ANMAT), on March 22, 2022.<br>Samples of the surveillance strategy implemented by the Ministry of Health of the Province of Buenos Aires that were used, were obtained from individuals that gave a written informed consent after receiving a fully explanation of the nature and possible consequences of the study. The study was approved on February 3, 2022, by the Central Ethics Committee of Buenos Aires Province (Comité de ética central de la provincial de Buenos Aires).

Note that full information on the approval of the study protocol must also be provided in the manuscript.

Field-specific reporting

Please select the one below that is the best fit for your research. If you are not sure, read the appropriate sections before making your selection.

☒ Life sciences ☐ Behavioural & social sciences ☐ Ecological, evolutionary & environmental sciences

For a reference copy of the document with all sections, see nature.com/documents/nr-reporting-summary-flat.pdf

Life sciences study design

All studies must disclose on these points even when the disclosure is negative.

Sample size	80 subjects ARVAC CG study and 18 subjects from the COVID-19 serology surveillance strategy implemented by the Ministry of Health of the Province of Buenos Aires.<br>Statistical analyses to determine sample size was not performed, as it is a first in humans phase I clinical trial and was not required from regulatory agencies. The number of subjects included is the typical or higher needed for a phase I study and and allowed to explore the safety of the vaccine candidate to initiate phase 2/3 studies. In addition, immunogenicity data and some statistical differences between doses together with similar safety allowed to select a dose to be used in the following clinical trial.
Data exclusions	Of the 80 volunteers who received at least one intramuscular dose of ARVAC CG in the deltoid, three were excluded 28 days after the first dose of the vaccine due to impossibility to complete the protocol for personal reasons. Of the 77 remaining, 59 were inoculated with two 25-µg vaccine doses, and 18 with two 50-µg vaccine doses. One study participant was tested as SARS-CoV-2 positive in the fifth visit of the protocol (day 28 after first dose). The participant was completely asymptomatic and following protocol instructions the application of the second dose was delayed. While the safety data of this volunteer at 28 days and after the second booster are included, the immunogenicity data at day 28 and at later time points were excluded because the immunogenicity against the virus may shape the antibody response and lead to misinterpretation of the results.
Replication	All experiments were performed in technical triplicates or duplicates using validated assays. All attempts at replication were successful.
Randomization	No randomization was performed. It is a phase 1 study with no control arm. Known potential covariates are controlled by eligibility criteria. Additionally, stratified data analysis was carried out to manage covariates such as time the type of primary vaccination scheme, prior COVID or presence anti-N antibodies, time since last dose, sex.
Blinding	Investigators, and laboratory personnel involved in assays were blind to assignment until the end of the follow-up period.

Reporting for specific materials, systems and methods

We require information from authors about some types of materials, experimental systems and methods used in many studies. Here, indicate whether each material, system or method listed is relevant to your study. If you are not sure if a list item applies to your research, read the appropriate section before selecting a response.

Materials & experimental systems

n/a	Involved in the study	
☐	☒	Antibodies
☐	☒	Eukaryotic cell lines
☒	☐	Palaeontology and archaeology
☒	☐	Animals and other organisms
☐	☒	Clinical data
☒	☐	Dual use research of concern
☒	☐	Plants

Methods

n/a	Involved in the study	
☒	☐	ChIP-seq
☒	☐	Flow cytometry
☒	☐	MRI-based neuroimaging

Antibodies

Antibodies used	Anti-Spike and anti-RBD IgG were determined by the use of comercial available ELISA kits. COVIDAR IgG kit was used to determine the anti-Spike antibody levels in plasma samples. Anti-RBD antibodies were determined by the DRG SARS-CoV-2 RBD IgG ELISA kit. Anti-N IgG antibody titers were determined by an In House developed ELISA using a Peroxidase-conjugated AffiniPure Goat Anti-Human IgG (H+L) (minimal cross-reaction to Bovine, Horse, and Mouse Serum Proteins) from Jackson ImmunoResearch Laboratories Ink (Code: 109135-088, Lot number:135723), final dilution 1:8000.
Validation	Comercial ELISA kits were validated by the manufacturers. Each ELISA plate included negative and positive controls. The run test were considered valid provided the control values and ranges given on the data sheet were met. In house anti-N Elisa was validated using positive samples and prepandemic serum samples. Each plate included positive and negative controls.

Eukaryotic cell lines

Policy information about cell lines and Sex and Gender in Research

Cell line source(s)	VERO E6 cells, source ATCC. VERO C1008 [Vero 76, clone E6, Vero E6] is a cell line exhibiting epithelial morphology that was isolated from the kidney of an African green monkey. This line is a clone of VERO 76 (ATCC CRL-1587).
Authentication	A cell bank was generated at INBIRS from the cells obtained from ATCC. The Cell line is in continuous use and used for SARS-CoV-2 neutralization assays performed at INBIRS. Cells are tested to be adherent and show some degree of contact inhibition and are suitable for supporting the growth of SARS-CoV-2 strains and other viral strains used in the institute.

Mycoplasma contamination	The Vero E6 cell line tested negative for mycoplasma contamination. Mycoplasma testing is routinely performed.
Commonly misidentified lines (See ICLAC register)	None.

Clinical data

Policy information about clinical studies

All manuscripts should comply with the ICMJE guidelines for publication of clinical research and a completed CONSORT checklist must be included with all submissions.

Clinical trial registration	The trial was prospectively registered in PRIISA.BA (Registration Code 6564) and in ANMAT. Registration was performed on February 2022, before the study beginning and before enrollment of the first participant. Also the same protocol with no changes was retrospectively registered on December 23, 2022 in ClinicalTrials.gov (NCT05656508).
Study protocol	The full trial protocol can be accessed in the supplementary material of this work.
Data collection	The trial was conducted at Clinical Pharma (Clínica CIAREC, Intense Life S.A, Buenos Aires, Argentina). Data were collected in a eCRF from Castor EDC.
Outcomes	Safety was the primary endpoint of the study.<br>Safety was assessed according to the scheme established in the Guidance for Industry, Toxicity Grading Scale for Healthy Adult and Adolescent Volunteers Enrolled in Preventive Vaccine Clinical Trials. Solicited local and systemic adverse events (AE) were recorded during the first 7 days after each dose of vaccine received, unsolicited events were recorded during the first 28 days after vaccination, laboratory tests were carried out after 7 and 28 days of each dose. The following symptom grading was used for local and systemic AE: grade 1 (mild) to grade 4 (potentially life-threatening). All safety information collected was available to an external Independent Committee of Data Review for continuous monitoring of any relevant AE and recommendations of modifying or interrupting the protocol as necessary.<br>Immunogenicity was the secondary endpoint. Outcomes were neutralizing antibodies against the Ancestral strain and the Gamma, Delta, Omicron BA.1 and Omicron BA.5 variants of concern of SARS-CoV-2, binding antibodies and cellular immune responses.
