# Supplementary figures and images for: Safety and immunogenicity of a SARS-CoV-2 Gamma variant RBD-based protein adjuvanted vaccine used as booster in healthy adults
Source: Nat Commun. 2023 Jul 28;14:4551. doi: 10.1038/s41467-023-40272-3 (PMC10382514; doi:10.1038/s41467-023-40272-3)

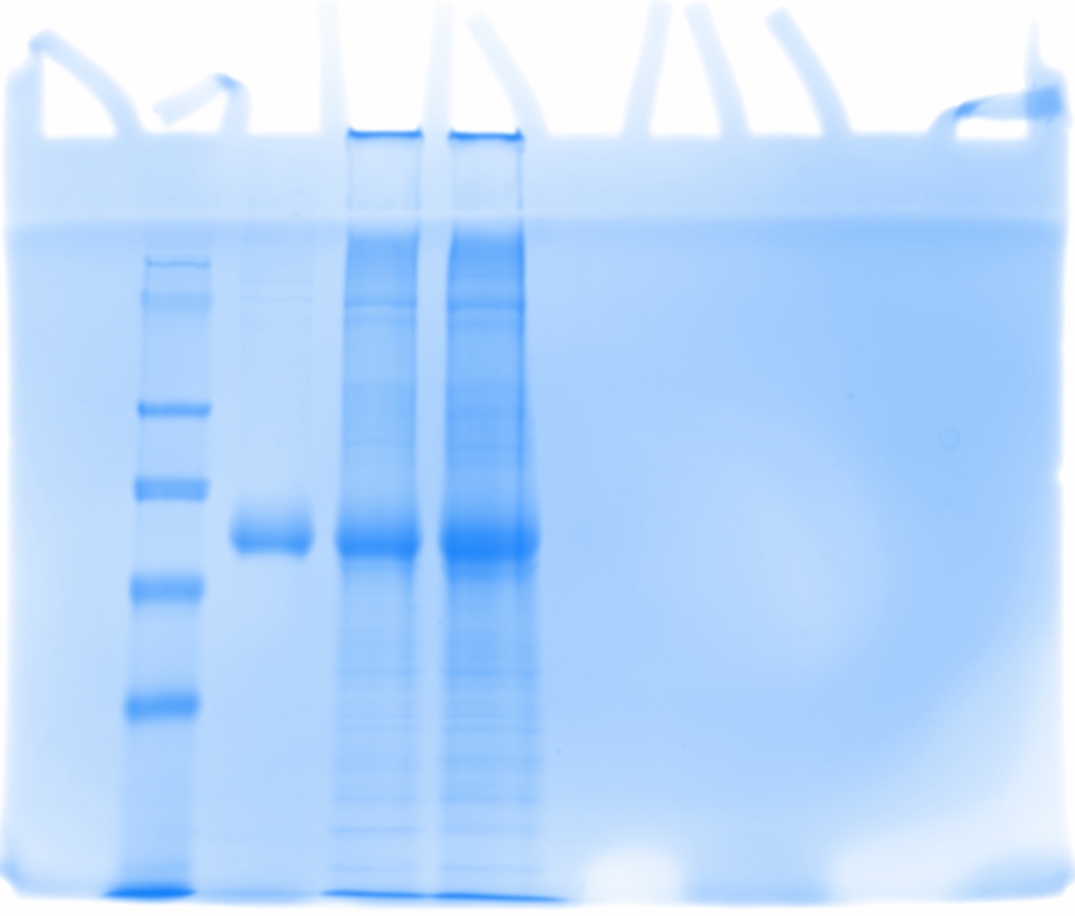

Supplement: Supplementary file 4 — Source Data [file 41467_2023_40272_MOESM4_ESM.zip › Source Data file/Supplementary Figure 11.jpg]

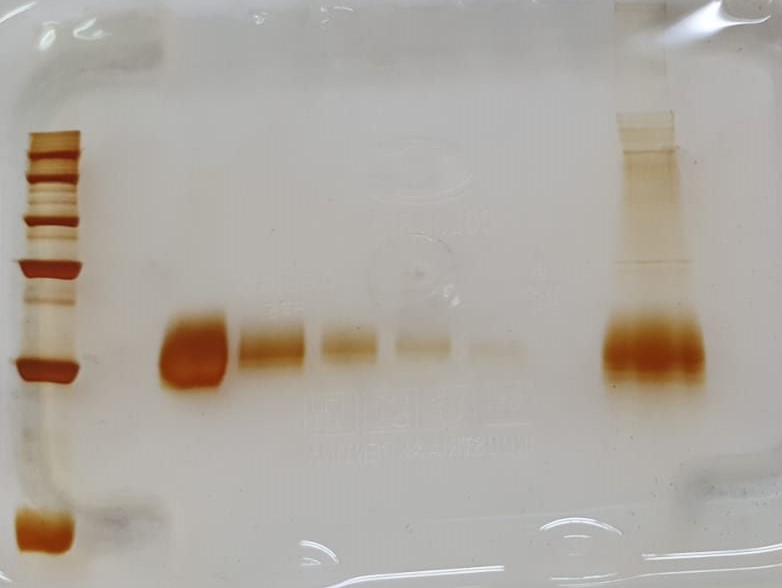

Supplement: Supplementary file 4 — Source Data [file 41467_2023_40272_MOESM4_ESM.zip › Source Data file/Supplementary Figure 12A.jpg]

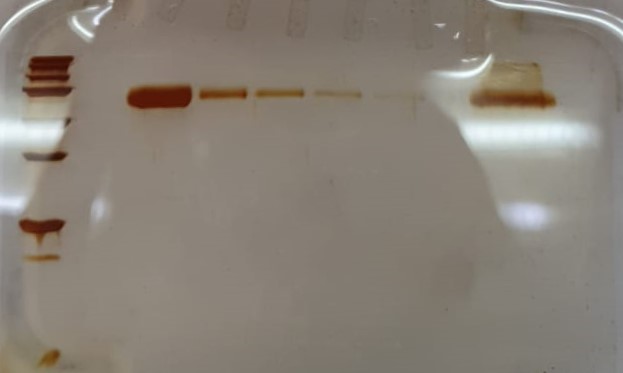

Supplement: Supplementary file 4 — Source Data [file 41467_2023_40272_MOESM4_ESM.zip › Source Data file/Supplementary Figure 12B.jpg]
